# Proteomic basis of mortality resilience mediated by *FOXO3* longevity genotype

**DOI:** 10.1007/s11357-023-00740-6

**Published:** 2023-03-07

**Authors:** Timothy A. Donlon, Brian J. Morris, Randi Chen, Eunjung Lim, Eric K. Morgen, Kristen Fortney, Naisha Shah, Kamal H. Masaki, Bradley J. Willcox

**Affiliations:** 1 Department of Research, NIH Center of Biomedical Research Excellence for Clinical and Translational Research on Aging, Kuakini Medical Center, Honolulu, Hawaii 96817 USA; 2 Department of Cell and Molecular Biology, John A. Burns School of Medicine, University of Hawaii, Honolulu, Hawaii USA; 3 Department of Geriatric Medicine, John A. Burns School of Medicine, University of Hawaii, Honolulu, Hawaii USA; 4 School of Medical Sciences, University of Sydney, Sydney, New South Wales Australia; 5 Department of Quantitative Health Sciences, John A. Burns School of Medicine, University of Hawaii, Honolulu, Hawaii USA; 6 BioAge Labs Inc., 1445A S 50th St, Richmond, California USA

**Keywords:** FOXO3, Genetics, Disease resilience, Cytokines, Inflammation, Cardiometabolic disease, Bone morphogenic protein signaling

## Abstract

FOXO3 is a ubiquitous transcription factor expressed in response to cellular stress caused by nutrient deprivation, inflammatory cytokines, reactive oxygen species, radiation, hypoxia, and other factors. We showed previously that the association of inherited *FOXO3* variants with longevity was the result of partial protection against mortality risk posed by aging-related life-long stressors, particularly cardiometabolic disease. We then referred to the longevity-associated genotypes as conferring “mortality resilience.” Serum proteins whose levels change with aging and are associated with mortality risk may be considered as “stress proteins.” They may serve as indirect measures of life-long stress. Our aims were to (1) identify stress proteins that increase with aging and are associated with an increased risk of mortality, and (2) to determine if *FOXO3* longevity/resilience genotype dampens the expected increase in mortality risk they pose. A total of 4500 serum protein aptamers were quantified using the Somalogic SomaScan proteomics platform in the current study of 975 men aged 71–83 years. Stress proteins associated with mortality were identified. We then used age-adjusted multivariable Cox models to investigate the interaction of stress protein with *FOXO3* longevity-associated *rs12212067* genotypes. For all the analyses, the *p* values were corrected for multiple comparisons by false discovery rate. This led to the identification of 44 stress proteins influencing the association of *FOXO3* genotype with reduced mortality. Biological pathways were identified for these proteins. Our results suggest that the *FOXO3* resilience genotype functions by reducing mortality in pathways related to innate immunity, bone morphogenetic protein signaling, leukocyte migration, and growth factor response.

**Supplementary Information:**

The online version contains supplementary material available at 10.1007/s11357-023-00740-6.

## Introduction

Considerable contemporary research is being devoted to increasing lifespan and, in particular, improving health-span, defined as the number of years free of disease. The number of years living with a chronic disease (YLCD) could therefore be referred to as “morbidity-span.” The latter is a relatively under-investigated area. YLCD has a major impact on public health and medical systems since these are the years of life that consume the most healthcare resources. At present, YLCD cannot be reliably anticipated. Being able to predict the length of YLCD would offer better allocation of resources to improve population health. This could include further resources devoted to strategies that increase biological resilience (currently under-leveraged), and spending more money treating or preventing disease in people predicted to have high YLCD, since this will have high impact at a population level. Understanding the biological pathways may permit therapies for those morbidities for which the patient is most at risk.

Aging is accompanied by deterioration of bodily structures and function. The capacity of organisms to withstand the impact of stress-induced damage is referred to as “resilience.” Possession of such an ability is likely to result in a greater number of years free of disease (i.e., improved health-span), but could also reduce mortality in those with disease (i.e., increase YLCD). Heritable genetic variants in only two genes, namely, *APOE* [1] and *FOXO3* [2] (see review: [3]), have been consistently associated with longevity in multiple populations globally.

Please note that, herein, *FOXO3* (in italics) refers to the gene, whereas FOXO3 (in plain text) refers to the protein, “FOXO” refers to both FOXO1 and FOXO3 proteins, *Foxo3* is the mouse version of the gene, *dFOXO* is the Drosophila version of the gene, and *daf16* is the *Caenorhabditis elegans* version of the gene.

In a previous study, we found that longevity associated *FOXO3* single nucleotide polymorphisms (SNPs) consisted of a group of variants in close genetic proximity (within a haplotype block) [4]. In that study, we identified at least 14 putative functional sequence variants that were predicted to modify transcription factor binding efficiencies and therefore had the potential to modulate *FOXO3* mRNA transcription. In the present study, we focus on the SNP *rs12212067*. *FOXO3* longevity-associated SNPs were associated with mitigation of the lifespan-shortening effect of having a cardiometabolic disease (CMD, i.e., one or more of the conditions coronary heart disease (CHD), hypertension, and type 2 diabetes) in late life [5]. This was an example of an effect on cellular and organismal resilience, whereby *FOXO3* genotypes did not prevent chronic disease, but, rather, extended the years of morbidity associated with having one or more of these conditions (YLCD).

FOXO proteins play important roles in many cellular processes, including glucose and lipid metabolism, apoptosis, autophagy, cell cycle inhibition, stress resistance, DNA repair, angiogenesis, inflammation, immune response, pluripotency, and differentiation. FOXO transcription factors are thought to protect cells from these insults and to assist in repair or elimination of damaged cells. These functions of FOXO proteins likely underlie their impact on longevity. In contrast, the deregulation of FOXO proteins has been shown to be involved in several diseases owing to their roles in autophagy [6]. FOXOs are, moreover, tumor suppressors and have other functions that may contribute to healthy aging (see review: [7]).

FOXOs impact cardiovascular disease through the maintenance of cardiomyocyte function in health and in pathological conditions such as hyperglycemic and ischemic stress [8]. FOXO proteins have been shown to inhibit and reverse cardiac hypertrophy, as observed in heart failure, through maintenance of a quiescent state and the promotion of apoptosis after mechanical stress-induced hypertrophy [9–12]. FOXO proteins are involved in the pathogenesis of type 2 diabetes. The insulin pathway senses the nutritional status of an organism and FOXO transcription factors relay this information to specific transcriptional targets. Therefore, FOXO proteins are considered as metabolic master regulators that control the response to nutrient availability [13].

FOXO proteins play critical roles in dementia as they are responsible for the maintenance of quiescence of neuronal stem cells and the clearance of reactive oxygen species (ROS) [14]. In a cellular model of Huntington disease, the co-expression of wild-type FOXO1 with mutant huntingtin protein promoted autophagy and clearance of the aberrant protein [15]. In addition, levels of nuclear FOXO3 were found to be increased in cells homozygous for Huntington disease mutation [14].

A more complete understanding of the pathways by which FOXO3 exerts its resilience effects to help promote longevity should provide better targets for aging intervention and a reduction in late-life morbidity. Multiple stress signaling pathways converge on the protein homeostasis network of cells. Activation of the integrated stress response during aging involves increased expression of stress response genes whose encoded proteins affect pathways capable of slowing the aging processes [16]. Deregulation in aging-related diseases leads to earlier mortality. From mechanistic, therapeutic, and financial perspectives, it would be valuable to discern pathways that differentiate “healthy” from “unhealthy” aging, namely, avoidance of, as compared to resilience against, chronic disease. There is abundant literature now that shows changes in serum concentration of particular proteins with aging, and that some of these are proteins associated with increased mortality. A prime aim of geroscience is to identify biomarkers of aging and implement this knowledge to reduce the burden of aging-related diseases, slow functional decline, and promote healthy aging [17]. We believe that proteins associated with increased mortality may be used as surrogates/biological markers for life-long stress, permitting us to examine and perhaps separate the effects of longevity/resilience genotypes of *FOXO3*, for example, on mortality in individuals experiencing chronic disease–related stress.

The present analysis was conducted as part of the Kuakini Hawaii Lifespan Study (KHHP) [18] and the Kuakini Hawaii Asia Aging Study (KHAAS) [2, 18–20], an embedded cohort study of healthy aging drawn from the original KHHP-KHAAS population. The KHHP cohort is robust for phenotype–genotype associations since the data collection was exceptionally accurate and involved cross-validation utilizing an expert Morbidity and Mortality Committee. The Japanese population in Hawaii is of Japanese origin, with little outbreeding and, based on the authors’ unpublished data, exhibits a smaller degree of genetic diversity than the overall population of Japan. The relatively high degree of genetic homogeneity made our cohort ideal for phenotype–genotype discovery.

In the present study, we use proteomic analyses in a population, followed for over five decades, to ascertain changes in concentrations of serum proteins with aging. In an attempt to better understand the mechanisms of action of the FOXO3 stress-response protein, we were particularly interested in those proteins that increase with biological age and have a propensity to increase risk of mortality. We refer to these as “stress proteins.” Our aims were (1) to identify proteins whose serum levels increase in old age and that are associated with increased risk of mortality and (2) to determine the association, if any, of these proteins with *FOXO3* genotype and mortality in those at increased risk (i.e., in the upper tertile of high-risk protein levels). We hypothesized that this study would identify pathways in which *FOXO3* genotype may influence not only longevity and healthy aging (i.e., avoidance), but also unhealthy aging (i.e., resilience) imposed by chronic diseases (YLCD). Such information should prove valuable to public health and provide more accurate estimates for the allocation of funds for late-life resource needs, such as Medicaid, and long-term care. Identification of druggable pathways may provide more focused therapeutic relief to those at the greatest risk.

## Methods

### Study cohort

Participants were American men of Japanese ancestry living on the island of Oahu, Hawaii. The men were recruited in 1965–1968 from World War II Selective Service records for the Kuakini Honolulu Heart Program (KHHP) [18], which continued from 1991 onwards as the Kuakini Honolulu-Asia Aging Study (KHAAS) [2, 18–20]. The present analysis was conducted as part of the Kuakini Hawaii Lifespan Study and the Kuakini Hawaii Health-span Study, an embedded cohort study of healthy aging drawn from the original KHHP-KHAAS population. Subjects had parents who were almost all from a limited geographic area of Japan, mostly the western, central, and southern regions [18, 21]. Recruitment took place at the same time and place (Oahu), meaning there was no apparent reason why genetic background should be substantially different among subjects. The KHHP cohort is robust for phenotype–genotype associations since the data collection was exceptionally accurate and involved cross-validation utilizing an expert Morbidity and Mortality Committee. The Japanese population in Hawaii is of Japanese origin, with little outbreeding and, based on the authors’ unpublished data, exhibits a smaller degree of genetic diversity than the overall population of Japan.

The demographic characteristics of subjects were as described previously [2, 22]. After recruitment in 1965–1968, subjects in the cohort had been followed with regular examinations and blood work until 2020, or death up to the end of 2021. Of 8006 men, 7965 had died (mean age at death 89.0 ± 6.2 SD years; range 72–108 years), and 36 (1%) were still alive (mean age 101.6 ± 1.9 SD years; range 100–108 years). Archived phenotypic data and blood samples from Examination 4 of the KHHP (1991–1993) were used. For the present study, we randomly selected 1000 subjects from among those men in KHHP examination 4 who were aged 71–83, and who also participated in KHHP examination 3 (in 1971–1974), and were, at that time, free from prevalent chronic diseases such as diabetes, kidney disease, liver disease, CHD, stroke, and chronic obstructive pulmonary disease.

Procedures performed were in accord with institutional guidelines and were approved by the Institutional Review Board of Kuakini Medical Center. Written informed consent was obtained at each examination from all study participants or from family representatives, if participants could not provide consent.

### Genotyping

Leukocyte DNA obtained from participants underwent genotyping of *FOXO3* SNP *rs12212067* by allelic discrimination assays using TaqMan (Applied Biosystems, Inc.) and a Life Technologies QuantStudio 12K Flex OpenArray system. The longevity-associated minor (*G*) allele of *rs12212067* creates a myeloid zinc finger 1 transcription factor binding site [22, 23]. Details of other SNPs in linkage disequilibrium with *rs12212067* and their predicted effect on transcription factor binding appear in Fig. S1.

### Proteomics methods

Protein concentrations in fasting serum were measured using the SOMAScan assay platform (SomaLogic Inc.). SOMAScan is an aptamer-based assay allowing for the simultaneous measurement and quantification of thousands of individual proteins. The assay uses modified aptamers, which are DNA strands created to have a 3D conformation so that each selectively recognizes a specific protein, to measure relative protein abundance via fluorescence readings from microarrays [24].

Samples selected for proteomic profiling were distributed across 12 assay plates, each of which accommodates 96 samples. Each plate includes, in addition to the cohort samples, special standardized samples for calibration and quality control as follows: three with buffer only (no sample added), five calibrator samples, and three quality control samples. Buffer-only samples are used to establish a baseline level of signal for each aptamer representing the absence of protein. Calibrator samples are standardized samples added to all plates and used for correction of systematic differences across plates.

Raw data were normalized using a probabilistic model taking into account sample-specific effects, dilution group effects, and plate effects, and which uses hybridization control sequences and calibrator samples [24] to estimate these effects. Modeling was performed using Gaussian distributions on log-transformed data, with inference using Markov-Chain Monte Carlo method.

### Statistical analyses

Proteins that were increased with age were determined using a linear regression model. The false discovery rate (FDR) method was applied for multiple testing [25]. A protein that increases with age was classified according to stress status, namely, “stressed” if the protein level was greater than or equal to its upper tertile and “non-stressed” if otherwise. Then, the proteins (“stresses”) were screened for their effects, adjusting for age effect on mortality in Cox proportional hazard models. Those proteins that showed as a risk factor for mortality adjusting for age were classified as stress proteins. To assess the *FOXO3* gene resilience effect of a stress protein, a Cox proportional hazard model was applied. All statistical analyses were performed using the Statistical Analysis System (SAS) version 9.4 [26]. Figures were generated using STATA 16 [27].

### Pathway analysis

An online pathway analysis package (GeneMANIA; http://genemania.org) was used to scan for significant FOXO3 interacting protein/gene pathways [28]. A brief description of their data sources can be found in Mostafavi et al. [28]. By default, the GeneMANIA prediction server uses one of two different adaptive network weighting methods. For longer gene lists, such as those used in the present study, GeneMANIA adopts the basic weighting method (called GeneMANIA^Entry-1^ in [28] and termed “assigned based on query genes” on its web site) and weights each network so that after the networks are combined, the query genes interact as much as possible with each other while interacting as little as possible with genes not in the list. GeneMANIA “learns” from longer gene lists, allowing a gene list–specific network weighting to be calculated.

## Results

### Final sample size and baseline variables

Of the 1000 men selected, 5 were excluded since we did not have complete proteomics data for these, and 20 were excluded since we did not have genotyping data for them in the final dataset. The sample size for the present study was thus 975. Baseline variables for *FOXO3 rs12212067 TT* and *TG*/*GG* genotypes are shown in Table S1. During 28 years of follow-up, 956 (98%) men died. The median follow-up time was 12.7 years.

### Modification of mortality risk by *FOXO3* genotype on mortality-increased protein levels

We screened 4575 serum protein aptamers using the Somalogic SomaScan v4.1 proteomics platform. Of these, 317 proteins, passed FDR adjustment, showed increases with age, and 184 proteins classified as stress proteins were, after FDR adjustment, associated with increased mortality.

**Fig. 1:**
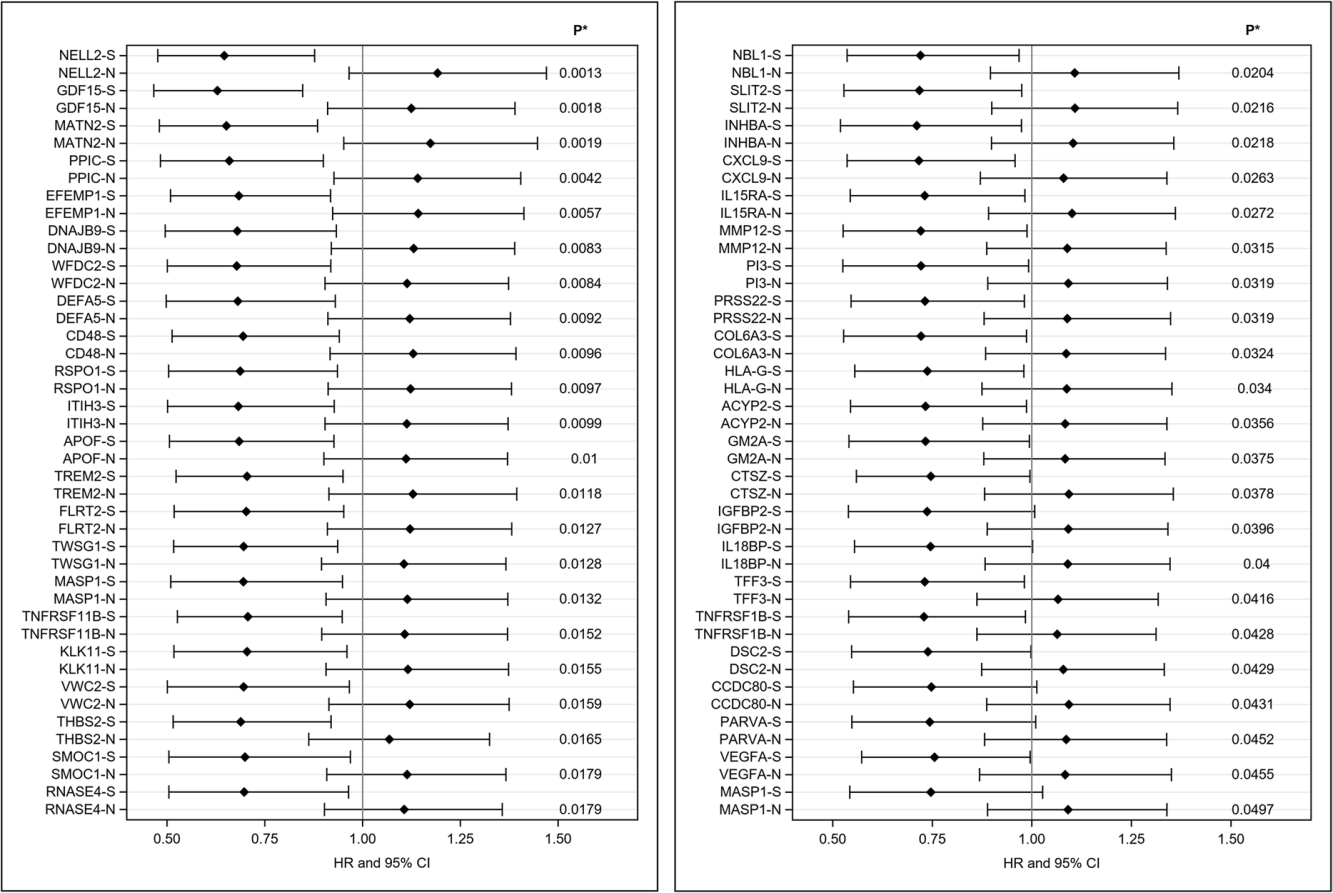
Forest plots of hazard ratios of contrasting *FOXO3* genotypes and mortality in stressed (S) and non-stressed (N) subjects for top 44 proteins that increase with aging. *p value for comparison of hazard ratios of *FOXO3* genotype (*FOXO3 rs12212067*) *G* allele carriers (*TG*/*GG*) vs. major allele homozygote (*TT*) with mortality in stressed and non-stressed subjects. “Stressed” refers to those subjects with upper tertile of mortality-associated proteins. Note: MASP1 (last entry in Table) is represented twice because two separate aptamers were identified

We then assessed *FOXO3* genotypes for potential reduction in mortality for these stress proteins in a multivariate Cox model adjusting for age, BMI, fasting glucose, smoking (pack-years), alcohol consumption (oz/month), and physical activity index. We refer to this reduction in expected mortality under conditions of stress versus no stress as “resilience,” and the proteins were termed as “FOXO3 resilience proteins.” We included the interaction term of *FOXO3* genotype with stress on mortality in the Cox model. When the interaction term was significant, it meant the effect of *FOXO3* genotype on mortality differed between “stressed subjects” compared with “non-stressed subjects.” To investigate the effect of FOXO3-associated resilience proteins, we compared *FOXO3 rs12212067* resilience genotypes (*TG* and *GG*) with the major allele homozygote, *TT*. In this way, we identified 44 *FOXO3* resilience proteins (Table 1, Fig. 1). Due to the nature of low power of the test for interaction, we did not perform multiple testing adjustment for the interaction terms. Table 1 and Fig. 1 show the hazard ratios and *p* values of the interaction between *FOXO3* genotype (*TG*/*GG* vs. *TT*) and stress status (stressed vs. non-stressed) for mortality.

**Table 1:** Effect of *FOXO3* genotype (*TG*/*GG* vs *TT*) on risk of mortality for aging-related proteins

**Protein/gene name**	**Gene symbol**	**UniProt ID**	**Stress**	p**	HR***	***p*** **for interaction**
Protein kinase C-binding protein NELL2	*NELL2*	Q99435	1	5.10E–03	0.647	1.30E–03
			0	1.00E–01	1.191	
Growth/differentiation factor 15	*GDF15*	Q99988	1	2.30E–03	0.629	1.80E–03
			0	2.80E–01	1.125	
Matrilin-2	*MATN2*	O00339	1	6.10E–03	0.652	1.90E–03
			0	1.30E–01	1.174	
Peptidyl-prolyl *cis*-*trans* isomerase C	*PPIC*	P45877	1	8.70E–03	0.659	4.20E–03
			0	2.10E–01	1.141	
EGF-containing fibulin-like extracellular matrix protein 1	*EFEMP1*	Q12805	1	1.10E–02	0.683	5.70E–03
			0	2.20E–01	1.142	
DnaJ homolog subfamily B member 9	*DNAJB9*	Q9UBS3	1	1.70E–02	0.68	8.30E–03
			0	2.40E–01	1.131	
WAP four-disulfide core domain protein 2	*WFDC2*	Q14508	1	1.20E–02	0.679	8.40E–03
			0	3.10E–01	1.114	
Defensin-5	*DEFA5*	Q01523	1	1.60E–02	0.681	9.20E–03
			0	2.80E–01	1.12	
CD48 antigen	*CD48*	P09326	1	1.80E–02	0.694	9.60E–03
			0	2.50E–01	1.13	
R-spondin-1	*RSPO1*	Q2MKA7	1	1.70E–02	0.687	9.70E–03
			0	2.80E–01	1.122	
Inter-alpha-trypsin inhibitor heavy chain H3	*ITIH3*	Q06033	1	1.50E–02	0.682	9.90E–03
			0	3.10E–01	1.113	
Apolipoprotein F	*APOF*	Q13790	1	1.40E–02	0.685	1.00E–02
			0	3.30E–01	1.111	
Triggering receptor expressed on myeloid cells 2	*TREM2*	Q9NZC2	1	2.20E–02	0.705	1.20E–02
			0	2.60E–01	1.129	
Leucine-rich repeat transmembrane protein FLRT2	*FLRT2*	O43155	1	2.30E–02	0.703	1.30E–02
			0	2.80E–01	1.121	
Twisted gastrulation protein homolog 1	*TWSG1*	Q9GZX9	1	1.70E–02	0.696	1.30E–02
			0	3.50E–01	1.106	
Sushi, von Willebrand factor type A, EGF, and pentraxin domain-containing protein 1	*MASP1*	Q4LDE5	1	2.20E–02	0.695	1.30E–02
			0	3.00E–01	1.115	
Tumor necrosis factor receptor superfamily member 11B	*TNFRSF11B*	O00300	1	2.00E–02	0.707	1.50E–02
			0	3.50E–01	1.108	
Kallikrein-11	*KLK11*	Q9UBX7	1	2.60E–02	0.705	1.50E–02
			0	3.00E–01	1.116	
Brorin	*VWC2*	Q2TAL6	1	3.00E–02	0.696	1.60E–02
			0	2.70E–01	1.121	
Thrombospondin-2	*THBS2*	P35442	1	1.10E–02	0.689	1.60E–02
			0	5.40E–01	1.069	
SPARC-related modular calcium-binding protein 1	*SMOC1*	Q9H4F8	1	3.20E–02	0.7	1.80E–02
			0	3.00E–01	1.114	
Ribonuclease 4	*RNASE4*	P34096	1	2.90E–02	0.697	1.80E–02
			0	3.30E–01	1.107	
			0	1.90E–01	0.87	
Neuroblastoma suppressor of tumorigenicity 1	*NBL1*	P41271	1	2.90E–02	0.72	2.00E–02
			0	3.40E–01	1.108	
Slit homolog 2 protein	*SLIT2*	O94813	1	3.30E–02	0.717	2.20E–02
			0	3.30E–01	1.109	
Inhibin beta B chain	*INHBA*	P09529	1	3.40E–02	0.711	2.20E–02
			0	3.50E–01	1.104	
C-X-C motif chemokine 9	*CXCL9*	Q07325	1	2.40E–02	0.717	2.60E–02
			0	4.80E–01	1.08	
Interleukin-15 receptor subunit alpha	*IL15RA*	Q13261	1	3.80E–02	0.731	2.70E–02
			0	3.70E–01	1.101	
Macrophage metalloelastase	*MMP12*	P39900	1	4.20E–02	0.721	3.20E–02
			0	4.20E–01	1.089	
Elafin	*PI3*	P19957	1	4.40E–02	0.722	3.20E–02
			0	4.00E–01	1.092	
Brain-specific serine protease 4	*PRSS22*	Q9GZN4	1	3.70E–02	0.732	3.20E–02
			0	4.30E–01	1.089	
Collagen alpha-3(VI) chain: bovine pancreatic trypsin inhibitor/Kunitz inhibitor domain, isoform 1	*COL6A3*	P12111	1	4.10E–02	0.721	3.20E–02
			0	4.30E–01	1.087	
HLA class I histocompatibility antigen, alpha chain G	*HLA-G*	P17693	1	3.60E–02	0.738	3.40E–02
			0	4.50E–01	1.088	
Acylphosphatase-2	*ACYP2*	P14621	1	4.00E–02	0.733	3.60E–02
			0	4.60E–01	1.084	
Ganglioside GM2 activator	*GM2A*	P17900	1	4.50E–02	0.733	3.80E–02
			0	4.50E–01	1.084	
Cathepsin Z	*CTSZ*	Q9UBR2	1	4.60E–02	0.746	3.80E–02
			0	4.20E–01	1.093	
Insulin-like growth factor-binding protein 2	*IGFBP2*	P18065	1	5.50E–02	0.737	4.00E–02
			0	4.10E–01	1.091	
Interleukin-18-binding protein	*IL18BP*	O95998	1	5.20E–02	0.745	4.00E–02
			0	4.20E–01	1.09	
Trefoil factor 3	*TFF3*	Q07654	1	3.70E–02	0.7312	4.20E–02
			0	5.60E–01	1.066	
Tumor necrosis factor receptor superfamily member 1B	*TNFRSF1B*	P20333	1	3.90E–02	0.729	4.30E–02
			0	5.70E–01	1.064	
Desmocollin-2	*DSC2*	Q02487	1	4.80E–02	0.739	4.30E–02
			0	4.80E–01	1.079	
Coiled-coil domain-containing protein 80	*CCDC80*	Q76M96	1	6.00E–02	0.748	4.30E–02
			0	4.00E–01	1.093	
Alpha-parvin	*PARVA*	Q9NVD7	1	5.80E–02	0.744	4.50E–02
			0	4.40E–01	1.086	
Vascular endothelial growth factor D	*VEGFA*	O43915	1	4.70E–02	0.755	4.60E–02
			0	4.80E–01	1.083	
Sushi, von Willebrand factor type A, EGF, and pentraxin domain containing protein 1	*MASP1*	Q4LDE5	1	7.30E–02	0.747	5.00E–02
			0	4.00E–01	1.091	

MASP1 is represented twice because two separate aptamers were identified

*Stress: 1 = upper tertile, 0 = lower/middle tertile

**p value for test of HR within stress status

***Hazard ratios were estimated from least square means from Cox proportional hazard model, adjusting for age, BMI, fasting glucose, smoking (pack-years), alcohol consumption (oz/month), and physical activity index

****p value for interaction term of *FOXO3* genotype and stress on mortality for comparison of hazard ratios of *FOXO3* genotype (*FOXO3 rs12212067 G* allele carriers, *TG*/*GG* vs. common allele homozygote *TT*) with mortality in stressed (1) and non-stressed (0) subjects

**Fig. 2:**
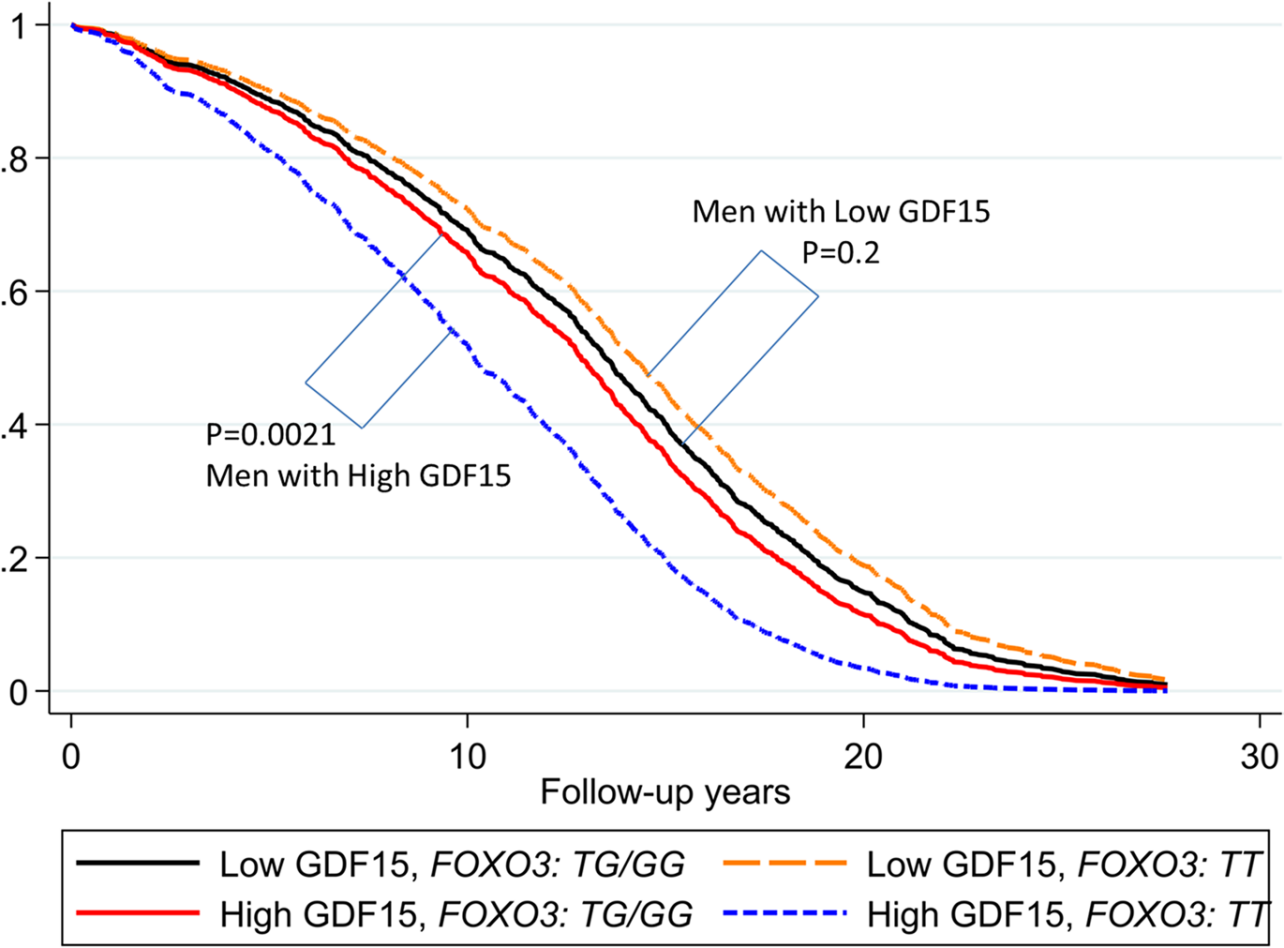
Survival curves for carriers of the *FOXO3* longevity (*G*) allele of SNP *rs12212067* comparing subjects with high vs. low GDF15 protein levels. Survival curves spanning the period from baseline (1991–1993) to December 31, 2019 for subjects with “Low” GDF15 and “High” GDF15 according to whether they were carriers of the *FOXO3* longevity-associated (*G*) allele or were major allele homozygotes (*TT*) of *FOXO3* SNP *rs12212067*. The survival probabilities were estimated from the Cox proportional hazard model (see “Methods” section): h(t) = h(t0)*exp(β1*Age + β2*BMI + β3*glucose + β4*High_GDF15 + β5*FOXO3_*G* + β6* (High_GDF15*FOXO3_*G*)) by fixing age at 75 years, BMI at the mean, 23.8 kg/m^2^, and glucose at the mean, 111 mg/dL (where β6 is the effect of the interaction of high GDF15 with *FOXO3* genotype (*G*-allele carriers vs. *TT* genotype) on mortality, giving (β6) = 0.0013). The *p* values for comparison of survival curves for the group with a Low GDF15 for *FOXO3*-*G* carriers vs. *FOXO3*-*TT* genotype, and comparison of survival curves for the group with a High GDF15 for *FOXO3 G*-allele carriers vs. *FOXO3 TT* genotype, were *p*=0.20 and *p*=0.0021, respectively. The *p* values for comparison of survival curves for *FOXO3*-*TT* genotype or for *FOXO3 G*-allele carriers for those with a Low GDF15 versus those with High GDF15 were *p* < 1.0 × 10^–7^ and *p* = 0.44, respectively

**Fig. 3:**
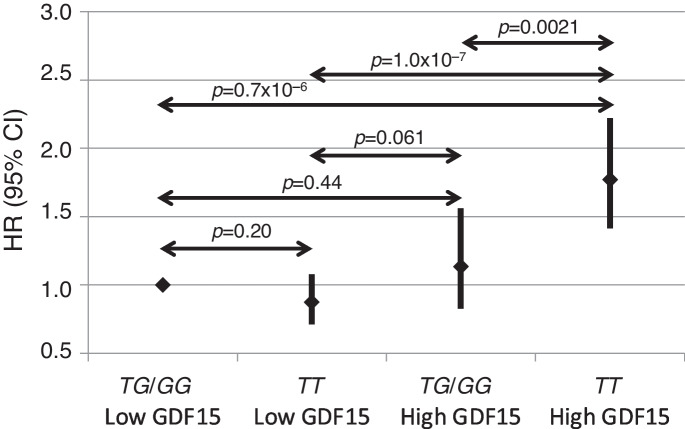
Mortality risk expressed as hazard ratio (HR) for subjects with low (lower/middle tertile) and high (upper tertile) GDF15 protein levels and *FOXO3* genotype. Subjects were grouped according to GDF15 level and *FOXO3* genotypes to compare their risks for mortality. HRs were estimated from Cox models adjusting for age, BMI and glucose level. This showed that the men under stress (with high GDF15) and with *FOXO3* longevity genotype (*TG*/*GG*) had: (1) similar risk for mortality as those not under stress (with low GDF15) regardless of *FOXO3* genotype (*p* = 0.44, *p* = 0.061); (2) significantly reduced risk for mortality compared to the men under stress (with high GDF15) and *FOXO3* genotype *TT* (*p *= 0.0021)

To demonstrate the effect of *FOXO3* genotype on mortality in the high-risk group in a more visual manner, we chose to plot survival curves for high and low levels of the well-established aging-related, mortality-associated protein, growth/differentiation factor 15 (GDF15) (Fig. 2). The mortality risk hazard ratios are shown in Fig. 3. The expected high risk of mortality for high GDF was greatly abated in carriers of the minor (*G*) allele of the longevity genotype of *rs12212067.* Figure S2 shows survival curves based purely upon the *FOXO3* genotype irrespective of circulating GDF15 levels.

The *FOXO3* genotype had a *direct* effect on the actual protein levels of only one protein, *CCDC80* (Table S2). CCDC80 is a coiled-coil domain-containing protein that is predicted to enable glycosaminoglycan binding activity. It may act upstream of or within extracellular matrix organization. It confers positive regulation of cell–substrate adhesion and response to bacteria, and is predicted to be located in the extracellular matrix.

### Biological pathways that mediate risk of *FOXO3* genotype on mortality

**Fig. 4:**
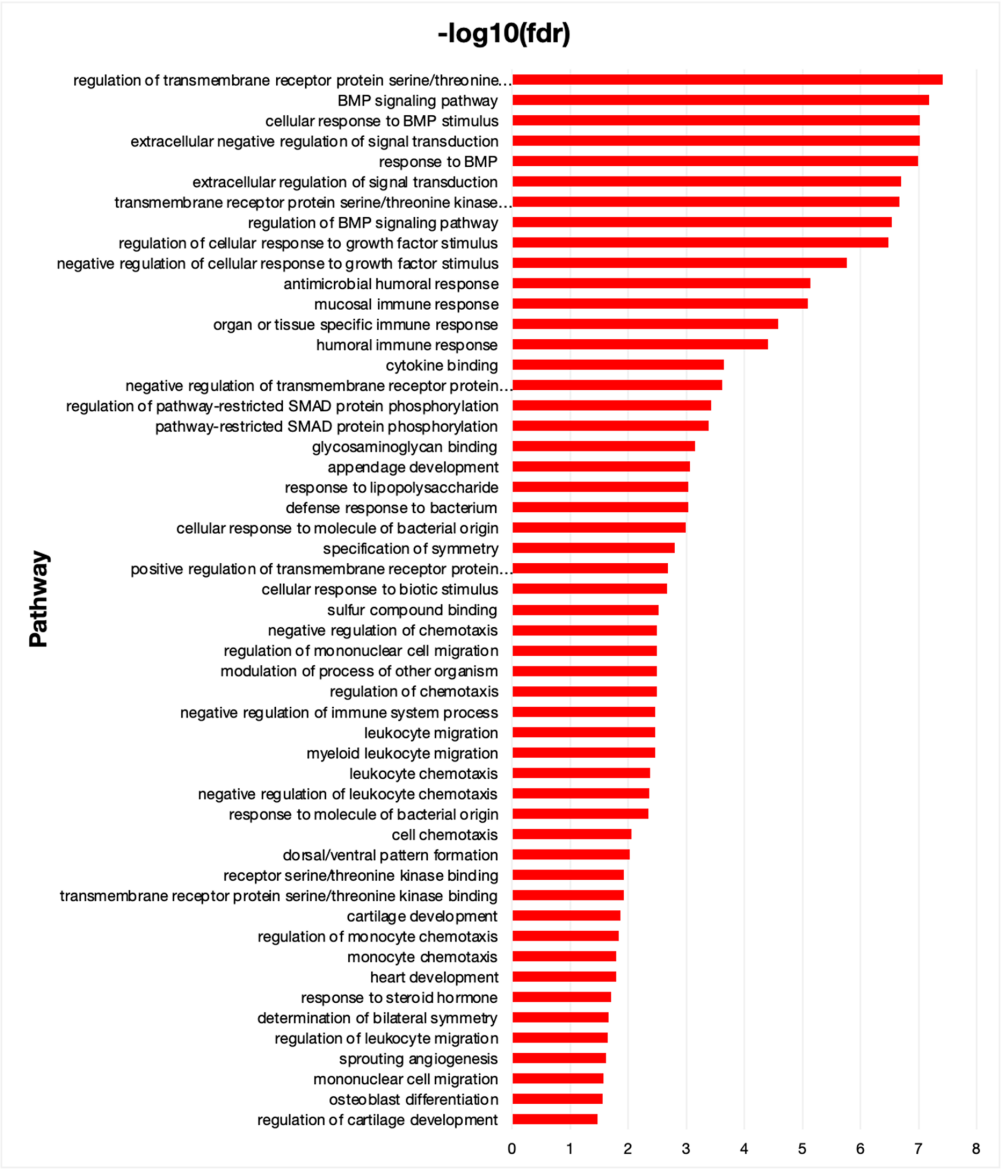
Biological pathways that mediate risk of *FOXO3* genotype on mortality. Aging-increased, mortality-increased, and *FOXO3* genotype–reduced risk (of mortality) genes (*n* = 43) were predicted to be involved in signal transduction, BMP signaling pathway, leukocyte migration, immune response, and growth factor response. A full list is available in Table 1 and Table S2

We used gene function prediction algorithms [28] to assess pathways involved in *FOXO3*-related mortality. Our analyses of FOXO3 gene/protein resilience identified various pathways, some of which had not been specifically ascribed to FOXO3 targets previously. Table 2 and Fig. 4 show that the resilience effect of *FOXO3* genetic variance relies ultimately on a combination of

1. Immune response
2. Bone morphogenic protein (BMP) signaling
3. Signal transduction
4. Leukocyte migration
5. Growth factor response.

**Table 2:** Predicted functions of genes for which *FOXO3* genotype reduces risk of mortality (age increased, mortality increased, risk decreased by genotype)

**Function**	**FDR**	**Genes in network**	**Genes in genome**
Antimicrobial humoral response	2.61E–15	13	97
Humoral immune response	4.22E–13	14	197
Defense response to bacterium	4.16E–12	12	135
BMP signaling pathway	2.29E–06	7	70
Cellular response to BMP stimulus	2.92E–06	8	122
Response to BMP	3.57E–06	8	128
Regulation of transmembrane receptor protein serine/threonine kinase signaling pathway	4.30E–06	9	201
Peptidase inhibitor activity	4.30E–06	8	135
Endopeptidase regulator activity	5.14E–06	8	141
Mucosal immune response	1.07E–05	5	26
Regulation of BMP signaling pathway	1.31E–05	6	59
Extracellular negative regulation of signal transduction	1.31E–05	4	10
Peptidase regulator activity	1.56E–05	8	170
Endopeptidase inhibitor activity	2.19E–05	7	115
Extracellular regulation of signal transduction	2.35E–05	4	12
Organ or tissue specific immune response	2.37E–05	5	33
Enzyme inhibitor activity	3.64E–05	9	281
Transmembrane receptor protein serine/threonine kinase signaling pathway	3.64E–05	9	282
Regulation of cellular response to growth factor stimulus	8.49E–05	8	222
Response to molecule of bacterial origin	5.73E–04	7	195
Response to lipopolysaccharide	1.08E–03	6	135
Cellular response to molecule of bacterial origin	1.22E–03	6	139
Negative regulation of cellular response to growth factor stimulus	1.35E–03	5	78
Cellular response to biotic stimulus	2.63E–03	6	161
Modulation of process of other organism	4.45E–03	5	101
Negative regulation of transmembrane receptor protein serine/threonine kinase signaling pathway	4.50E–03	5	102
Regulation of pathway-restricted SMAD protein phosphorylation	1.03E–02	4	58
Pathway-restricted SMAD protein phosphorylation	1.13E–02	4	60
Transmembrane receptor protein serine/threonine kinase binding	1.73E–02	3	23
Receptor serine/threonine kinase binding	1.73E–02	3	23
Appendage development	2.00E–02	4	71
Negative regulation of growth	3.47E–02	5	163
Specification of symmetry	3.47E–02	4	83
Positive regulation of transmembrane receptor protein serine/threonine kinase signaling pathway	4.24E–02	4	88
Cytokine binding	6.77E–02	4	100
Mesonephros development	8.54E–02	3	42
Mesonephric tubule development	8.54E–02	3	42
Mesonephric epithelium development	8.54E–02	3	42
Mesoderm development	8.93E–02	3	43

Possible gene association network and gene functions of the top 30 proteins/genes. In silico analyses were performed using the GeneMANIA (gene function prediction using a multiple association network integration algorithm)—an integrated interaction network program that predicts gene functions and possible interaction networks using many large publicly available datasets including protein–protein and genetic interaction networks

*FDR*, false discovery rate

Table S3 lists the top 35 resilience proteins/genes and their functions. Table 3 provides a more general model of *FOXO3* resilience. The literature indicates that FOXO3 is a stress-response protein implicated in ameliorating effects of ROS, radiation, endoplasmic reticulum stress, heat, mitochondrial dysfunction, starvation, hypoxia, NAD^+^/NADH imbalance, and inflammation (see review: [7]).

**Table 3:**
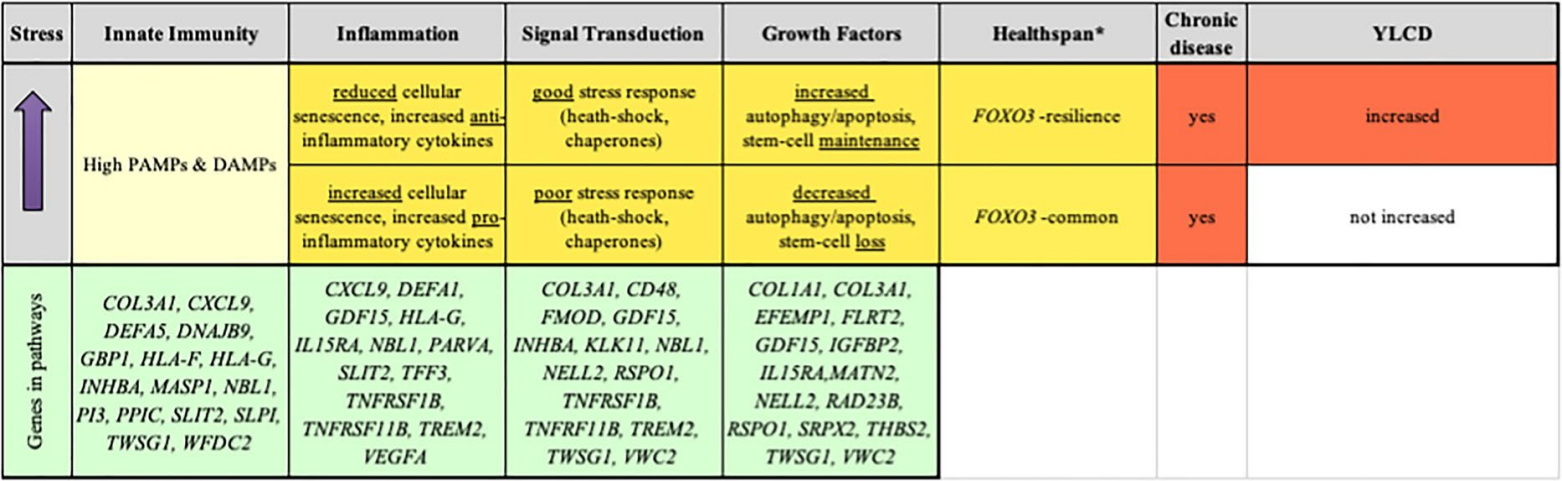
Proposed model for effect of *FOXO3* resilience genotype on mortality

Protein levels were assessed for age-related changes and increased mortality rates in order to identify proteins associated with long-term stress. High-stress proteins were identified and examined for effects involving the high-risk persons (upper tertile). Age-increased, genotype-reduced risk (of mortality) genes (*n* = 44) were predicted to be involved in signal transduction, BMP signaling pathway, leukocyte migration, immune response, and growth factor response. Key proteins/genes are listed below their predicted biological system, but may be included at multiple points. See Table 1 and Table S2 for the full list of proteins

*FOXO3 resilience genotype responds to stressful lifestyle leading to improved stress response and reduced mortality. *FOXO3* resilience genotype is a component of the longevity haplotype

*PAMPs*, pathogen-associated molecular pattern molecules; *DAMPs*, danger-associated molecular pattern molecules

## Discussion

### Newly discovered *FOXO3* pathways

Based on our findings, we propose a *FOXO3* resilience model using previous knowledge as well as the new data obtained here. The key factors involved in reducing mortality risk include the following:

1. Response to innate immunity: Increasing levels of cellular stress, resulting in moderating levels of danger-associated molecular patterns (DAMPs) and pathology-associated molecular patterns (PAMPs).
2. Reduced inflammation: The molecules involved serve to initiate formation of inflammasomes and other stress-response structures, resulting in release of inflammatory cytokines and activation of other signaling pathways.
3. Moderated signal transduction: Signaling pathways are implemented in order to respond to damaged organelles, cells, and extracellular structures and tissues.
4. Moderated growth factor levels: Damaged proteins are removed by autophagy and damaged cells are removed by apoptosis, thus increasing levels of growth factors necessary for cellular replacement and tissue repair via stem cell differentiation, without leading to stem-cell exhaustion.
5. Chronic disease: While chronic disease is not delayed, the *FOXO3* resilience genotype delays mortality by extending the functionality of those systems shown above.

The model in Table 3 includes those stress proteins that have been implicated in the present study, along with their roles in resilience. Note that a given protein and/or its gene may be present at multiple points in the model, a prime example being GDF15.

### *FOXO3* longevity versus resilience genotype

It should be noted that we found SNP *rs12212067* to be the only *FOXO3* variant of the eight longevity variants currently tested that showed a statistically significant association with stress-protein resilience. While this variant was in linkage disequilibrium with other longevity SNPs, the equilibrium is incomplete. We believe that carriers of the *rs12212067 *variant may represent resilience genotypes that are a component of the longevity haplotype which comprises at least 14 SNPs [4]. The longevity-associated haplotype frequency was much higher (0.24 vs. 0.09 (from dbSNP JPN)) than the “resilience genotype” (Fig. S1). The *rs12212067* resilience variant has previously been reported, by genome-wide association studies, to be associated with a milder course of Crohn’s disease and rheumatoid arthritis [29, 30]. The variant is associated with food preference/avoidance in Crohn’s disease patients. A neighboring SNP (*rs12196996*) has been reported to affect mRNA processing, including the expression of *circFOXO3*, a circular RNA associated with CHD risk [31].

New pathways identified in the current study include extracellular signal transduction, BMP signaling, leukocyte migration, and growth factor response. Extracellular signal transduction is a broad category of molecules traveling from one cell to another and most often involves protein phosphorylation, but may also include growth factors (e.g., transforming growth factor β, TGF-β), chemokines (e.g., interleukins), hormones (e.g., estrogen), survival factors (e.g., insulin-like growth factor 1, IGF1), and extracellular matrix proteins (e.g., integrin). At the top of the list of proteins modified by *FOXO3* genotype is neural epidermal growth factor-like like 2 (NELL2) that causes cell proliferation and inhibition of apoptosis when overexpressed in benign prostate hyperplasia [32]. NELL2 is, moreover, required for neuron survival through the modulation of MAPK pathways.

BMPs are a group of growth factors also known as cytokines and as metabologens that are members of the TGF-β family [33]. These proteins transduce their signals through type I and type II serine-threonine kinase receptors and their intracellular downstream effectors, including small mothers against decapentaplegic (SMAD) proteins [34]. Also, at the top of our list was GDF15, which cooperates with other proteins to mediate the innate immune response to bacterial lipopolysaccharide and to viruses [35]. GDF15 is considered an anti-inflammatory protein at low levels [36]. However, its levels are known to increase in chronic disease [37] and may be an indirect indicator of cellular stress [38]. GDF15 is a mitokine, where mitokines are soluble molecules produced in response to mitochondrial stress, immunosenescence, and inflammaging [39]. Mitokines respond to the increase in stress-related inflammatory cytokines induced by interleukins IL-1β and IL-2, TNF-α, and TGF-β, but may directly lower levels of IL-18 and IL-1β [40]. Studies in transgenic mice have shown that GDF15 lowers expression of pro-inflammatory cytokines IL-18, IL-1β, TNF-α, KC, IL-6, and MCP1 [41]. GDF15 is a secreted ligand of the TGF-β superfamily of proteins. Ligands of this family bind various TGF-β receptors leading to recruitment and activation of SMAD family transcription factors. GDF15 is expressed in a broad range of cell types, acts as a pleiotropic cytokine, and is involved in the stress response program of cells after cellular injury. Increased GDF15 levels are associated with disease states such as tissue hypoxia, inflammation, acute injury, and oxidative stress [39]. Both intracellular GDF15 and the circulating mature GDF15 are implicated in biological processes such as energy homeostasis and body weight regulation [41]. GDF15 is one of a number of biomarkers of frailty onset which together highlight the importance of inflammation and nutrient sensing in this condition [42]. Among GDF15’s related pathways are CREB and ERK signaling.

Leukocyte migration (extravasation) involves the passage of cells through the intact vessel wall and the movement of leukocytes out of the circulatory system and toward the site of tissue damage or infection as part of the innate immune response [43]. This process relies heavily on the maintenance of the extracellular matrix. Matrilin-2 (MATN2) [44] and EGF receptor (EGF)-containing fibulin-like extracellular matrix 1 (EFEMP1) are members in this pathway [45].

### Model of *FOXO3* mortality resilience

Our model of *FOXO3* mortality resilience (Table 3) includes four major biological systems in aging in which FoxO3 has been shown to be involved: (1) innate immunity, (2) inflammation (e.g., senescence-associated secretory phenotype (SASP)), (3) signal transduction, and (4) growth factor regulation and response (e.g., stem-cell maintenance, autophagy, and apoptosis). We have attempted to explain how the various stress-related proteins/genes fit into each of these systems. However, many of them play multiple roles and are involved in several pathways. The information on gene function presented below, but not the references, was taken largely from GeneCards [46] and is further detailed in Table S3.

#### Innate immunity

Innate immunity involves receptors, such as toll-like receptors (TLRs), heat-shock proteins, and high-mobility group proteins, that recognize protein patterns foreign to the cell and thereby present as invading pathogens [47]. These pathogenic (microbial)-associated molecular patterns (PAMPs) are small molecular motifs conserved within a class of microbes [48]. DAMPs are molecules within cells that are a component of the innate immune response released from damaged or dying cells due to trauma or an infection by a pathogen [49, 50]. Once a DAMP is released from the cell, it promotes a noninfectious inflammatory response by binding to a pattern-recognition receptor [50]. Inflammation is a key aspect of the innate immune response because it is used to help mitigate future damage to the organism by removing harmful invaders from the affected area and start the healing process [51]. In various model organisms, FoxO proteins modulate the innate immune system**,** including in mouse [52], *Drosophila* [53], *Caenorhabditis elegans* [54], and *Hydra* [55].

Foxo1 and 3 (DAF-16)-mediated immunity in adult *C. elegans* requires SMK-1, a regulatory subunit of the PP4 protein phosphatase complex [54]. The human homolog of SMK-1 is PPP4R3A, a component of the PP4 complex that dephosphorylates H2AX. Phosphorylation of the Ser-139 residue of the histone variant H2AX, forming γH2AX, is an early cellular response to the induction of DNA double-strand breaks [56] and is known to increase with age. dFOXO, the *Drosophila* equivalent of human FOXO3, promotes tolerance to hypoxia via the innate immunity transcription factor NF-κB/relish [57]. FOXO transcription factors are involved in the cellular responses to bacterial stimuli and act as central regulators of innate immune functions in respiratory epithelial cells [52].

In response to protein misfolding and endoplasmic reticulum (ER) stress, FOXO proteins integrate upstream ER stress and unfolded protein response (UPR) signals with the transcriptional machinery to decrease translation, promote cell survival/termination, and increase the levels of ER-resident chaperones and of ER-associated degradation (ERAD) components to restore ER homeostasis [58].

One of the stress proteins we identified was peptidase inhibitor 3 (PI3), which functions as an antimicrobial protein against Gram-positive and Gram-negative bacteria, and fungal pathogens. CCCL9 is part of a chemokine superfamily that encodes secreted proteins involved in immunoregulatory and inflammatory processes [59]. PI3 is thought to be involved in T-cell trafficking [60]. Defensins such as DEFA5 are a family of antimicrobial and cytotoxic peptides thought to be involved in host defense [61]. They are abundant in the granules of neutrophils [62] and are also found in the epithelia of mucosal surfaces such as those of the intestine, respiratory tract, urinary tract, and vagina. Defensins possess antimicrobial activity against Gram-negative and Gram-positive bacteria [63]. They are thought to kill microbes by permeabilizing their plasma membrane. DNAJB9 is localized within the endoplasmic reticulum, is induced by ER stress, and protects stressed cells from apoptosis [64]. Among its related pathways are metabolism of proteins and the UPR. DNAJB9 is required for survival of B-cell progenitors and for normal antibody production. MASP1 is a serine protease that functions as a component of the lectin pathway of complement activation. The complement pathway plays an essential role in the innate and adaptive immune response. PPIC is a peptidyl-prolyl *cis*-*trans* isomerase (PPIase) that catalyzes the *cis*-*trans* isomerization of proline imidic peptide bonds in oligopeptides and accelerate that this the folding of proteins.

#### Inflammation

The NLRP3 inflammasome is a critical component of the innate immune system [65]. It mediates caspase-1 activation and the secretion of proinflammatory cytokines IL-1β and IL-18 in response to microbial infection and cellular damage. However, the aberrant activation of the NLRP3 inflammasome has been linked to several inflammatory disorders, which include cryopyrin-associated periodic syndromes, Alzheimer’s disease, diabetes, and atherosclerosis [65]. FOXO3 restores autophagy flux and attenuates the activation of the NLRP3 inflammasome in Kupffer cells by promoting the transcription of Bim (BCL2L11) [66], suggesting that this could be a potential therapeutic target in non-alcoholic fatty liver disease and other obesity-related diseases. BCL2L11 induces apoptosis, possibly through a caspase-mediated pathway. FOXO3 [67] and FOXO4 [68] are effective inhibitors of NF-κB signaling and can reduce immune responses.

Cellular senescence is a process of permanent cell-cycle arrest during which cells are unable to re-enter the cell cycle despite the presence of growth factors, therefore limiting the lifespan of mammalian cells and preventing unlimited proliferation. Cellular senescence is beneficial during normal embryonic development and tissue damage because of its ability to promote tissue remodeling and renewal [69]. Overexpression of *FOXO3* suppressed the senescence process of cerebral microvascular endothelial cells (CMECs) under replicative stress by re-activating the transcription of antioxidant genes and thereby inhibiting ROS generation [70].

CXCL9 is part of a chemokine superfamily that encodes secreted proteins involved in immunoregulatory and inflammatory processes [71]. It is thought to be involved in T-cell trafficking. CXCL9 binds to C-X-C motif chemokine 3 and is a chemoattractant for lymphocytes but not for neutrophils. This cytokine affects the growth, movement, or activation state of cells that participate in immune and inflammatory response. HLA-G is a non-classical major histocompatibility class Ib molecule involved in immune regulatory processes at the maternal–fetal interface [72]. Upon interaction with KIR2DL4 and LILRB1 receptors on decidual NK cells, it triggers NK cell senescence–associated secretory phenotype as a molecular switch to promote vascular remodeling and fetal growth in early pregnancy. TNFRSF1B and TNFRSF11B are members of the TNF-receptor superfamily. Among related pathways are cytokine signaling in immune system and osteoblast signaling. Gene Ontology (GO) annotations include signaling receptor activity and cytokine activity. TREM2 functions in immune response and may be involved in chronic inflammation by triggering the production of constitutive inflammatory cytokines [73]. It regulates microglial proliferation by acting as an upstream regulator of the Wnt/beta-catenin signaling cascade. REM2 also regulates microglial chemotaxis and process outgrowth, and also the microglial response to oxidative stress and lipopolysaccharide. It suppresses PI3K and NF-κB signaling in response to lipopolysaccharide, thus promoting phagocytosis, suppressing pro-inflammatory cytokine and nitric oxide production, inhibiting apoptosis, and increasing expression of IL10 and TGFβ [74]. During oxidative stress, REM2 promotes anti-apoptotic NF-κB signaling and ERK signaling. It also has a role in microglial mTOR activation and metabolism.

#### Signal transduction, growth factors, and extracellular matrix

Signal transduction is a broad category of inter- and intra-cellular communications of molecular events commonly involving phosphorylation catalyzed by protein kinases. The most consequential pathway involving FOXO is insulin/IGF-1 signaling in which nutrients are sensed in a balance between proliferation versus quiescence. Activation of the PI3K/AKT pathways leads to the inhibition of FOXOs and activation of mTOR, thus regulating the cell cycle (see review: [75]). The PI3K/AKT pathways cause activation of FOXOs, leading to cell-cycle arrest and apoptosis [76]. The MAPK/ERK pathway plays an integral part in cell-cycle entry and proliferation by integrating mitogen and stress signals [77].

Inhibin subunit β E (INHBA) is an important paralog of the TGF-β gene. INHBA regulates food intake, energy expenditure, and body weight in response to metabolic and toxin-induced stresses. By binding to its receptor, GFRAL, GDF15 activates GFRAL-expressing neurons localized in the area postrema and nucleus tractus solitarius of the brainstem [78]. This then triggers the activation of neurons localized within the parabrachial nucleus and central amygdala, which constitutes part of the “emergency circuit” that shapes feeding responses to stressful conditions. In hepatocytes, GDF15 inhibits growth hormone signaling. Inhibins and activins are involved in regulation of a number of diverse functions such as hypothalamic and pituitary hormone secretion, gonadal hormone secretion, germ cell development and maturation, erythroid differentiation, insulin secretion, nerve cell survival, embryonic axial development, or bone growth, depending on their subunit composition [79].

Neuroblastoma, suppression of tumorigenicity 1 (NBL1) contains a domain resembling the C-terminal cystine knot-like (CTCK) motif found in a number of signaling molecules. These proteins are secreted and act as BMP antagonists by binding to BMPs and preventing them from interacting with their receptors [80].

Neuron-specific EGF-like protein 2 (NELL2) is a glycoprotein containing several von Willebrand factor C domains and EGF-like domains. Studies in mouse suggest that this protein plays a role in neural cell growth and differentiation as well as in oncogenesis and is required for neuron survival through the modulation of MAPK pathways [81].

The extracellular matrix harbors many of the wound-healing cells that include proteins associated with tissue growth and repair [82]. Cluster of differentiation 48 (CD48) is a neutrophil and pancreatic elastase-specific inhibitor. The protein COL6A3 (collagen α-3(VI) chain) contains von Willebrand Factor type A domains that explain its importance in organizing matrix components. EFEMP1 encodes a member of the fibulin family of extracellular matrix glycoproteins that contain tandemly repeated epidermal growth factor-like repeats. Among its related pathways are integrin pathway and ERK signaling. Its binding to EGFR induces EGFR autophosphorylation and the activation of downstream signaling pathways. Fibronectin leucine-rich repeat transmembrane 2 protein (FLRT2) has a role in fibroblast growth factor (FGF)-mediated signaling cascades [83]. It is required for normal organization of the cardiac basement membrane during embryogenesis, and for normal embryonic epicardium and heart morphogenesis. MATN2 is a member of the von Willebrand factor A domain containing protein family. R-spondin-1 (RSPO1) is a secreted activator protein that positively regulates the Wnt signaling pathway. In mice, this protein induces the rapid onset of crypt cell proliferation and increases intestinal epithelial healing, providing a protective effect against chemotherapy-induced adverse effects [84]. RSPO1 is a beta-cell growth factor and insulin secretagogue [85]. Thrombospondin-2 (THBS2) mediates cell-to-cell and cell-to-matrix interactions. This protein has been shown to function as a potent inhibitor of tumor growth and angiogenesis [86]. Twisted gastrulation protein homology 1 (TWSG1) enables TGF-β binding activity. It is involved in several processes, including BMP signaling [87], negative regulation of CD4-positive, α-β T-cell proliferation, positive regulation of pathway-restricted SMAD protein phosphorylation, and TGF-β receptor signaling pathway. THBS2 seems to antagonize BMP signaling by forming ternary complexes with CHRD and BMPs, thereby preventing BMPs from binding to their receptors. In addition to the anti-BMP function, it also has pro-BMP activity, partly mediated by cleavage and degradation of CHRD, which releases BMPs from ternary complexes. THBS2 may be an important modulator of BMP-regulated cartilage development and chondrocyte differentiation. Von Willebrand factor C containing 2 (WC2; also known as brorin) encodes a secreted BMP antagonist. Brorin is possibly involved in neural function and development [88], and may have a role in cell adhesion. BMP antagonist may play a role in neural development [89].

#### Autophagy

Autophagy controls cellular remodeling and quality control [90]. Dysregulated autophagy has been implicated in obesity, diabetes, cardiovascular disease, neurodegenerative diseases, and cancer. FOXO transcription factors have a multifaceted role in autophagy regulation and dysregulation [6]. Nuclear FOXOs transactivate genes that control the formation of autophagosomes and their fusion with lysosomes. Independently of transactivation, cytosolic FOXO proteins induce autophagy by directly interacting with autophagy proteins. Autophagy is also controlled by FOXOs through epigenetic mechanisms (see review: [3]). FOXO1 is primarily present in the cytosol, while FOXO3 is partitioned equally in both cytoplasm and nucleus.

In muscle, FOXO1 and FOXO3 elevate phagocytosis by increasing the expression of autophagy genes, mainly working as part of the core machinery, and additionally increase protein degradation via the proteasomal pathway [6]. In particular, FOXO3 increases the capacity of the lysosome to degrade incoming cargo, indicating a role for lysosomal function in muscle atrophy [91]. UV excision repair protein RAD23B is a multiubiquitin chain receptor involved in modulation of proteasomal degradation [92]. It may play a role in endoplasmic reticulum–associated degradation (ERAD) of misfolded glycoproteins by association with PNGase and delivering deglycosylated proteins to the proteasome [93, 94].

#### Apoptosis

Apoptosis is a form of programmed cell death that occurs in multicellular organisms [95]. It can be initiated through one of two pathways. In the intrinsic pathway, the cell kills itself because it senses cell stress, while in the extrinsic pathway the cell kills itself because of signals from other cells. Both pathways induce cell death by activating caspases, which are proteases or other enzymes that degrade proteins. Excessive apoptosis causes atrophy, whereas an insufficient amount results in uncontrolled cell proliferation, such as autoimmune diseases and cancer [96, 97]. Interleukin 15 receptor α subunit (IL15RA) encodes a cytokine receptor that specifically binds interleukin 15 (IL15) with high affinity and that is reported to enhance cell proliferation and expression of apoptosis inhibitor BCL2L1/BCL2-XL and BCL2 [98].

#### Stem cell maintenance

Stem cells are undifferentiated or partially differentiated cells that can differentiate into various types of cells and proliferate indefinitely to produce more of the same stem cell through a process of self-renewal. FOXO1 and FOXO3 have been reported to regulate germ cells, neural stem cells, hematopoietic stem cells, muscle satellite cells, and cancer stem cells [99].

FOXO3 serves as a core regulator of cellular homeostasis, stress response, and longevity through its ability to modulate a variety of stress responses during nutrient shortage, oxidative stress, hypoxia, heat shock, and DNA damage [100–103]. By reducing oxidative damage responsible for aging, FOXO3-mediated responses to stress are pivotal to health-span and lifespan [104]. Depending on the stress stimulus and subcellular context, once activated, FOXO3 can induce specific sets of nuclear genes, including cell-cycle inhibitors, pro-apoptotic genes, scavengers of ROS, autophagy effectors, and gluconeogenic enzymes [103]. On the other hand, under glucose restriction, FOXO3 translocates to mitochondria to stimulate transcription of oxidative phosphorylation genes, thus restoring cellular ATP levels [103]. FOXO3 target genes and the pathways that their gene products serve are diverse and sometimes antagonistic, meaning FOXO3 is an adaptable player in the dynamic homeostasis of normal and stressed cells [103].

FOXO proteins are critical in response to cellular damage, and help to orchestrate autophagy, the process of marking old proteins/structures, and implementing apoptosis and cellular death, when damage is insurmountable and cannot be corrected [6]. FOXO transcription factors have been shown to regulate metabolic homeostasis, neurogenesis and neuroprotection, cardiac remodeling, skeletal muscle homeostasis, immunity, endocytosis, stem cell homeostasis, and cancer cell growth and invasion [6].

FOXO proteins play pivotal roles in determining whether there are sufficient nutrients for growth or whether cells remain quiescent [105]. It is not clear whether long-lived individuals are born with more stem cells, which may cause them to have a higher prevalence of cancer, or are more adept at maintaining the stem cells they are born with. FOXO3 has been shown to be critical in the maintenance of adult hematopoietic stem cells [99]. It has been found to be the main transcription factor regulating autophagy-related gene expression and pathways in hemopoietic stem cells [106], has been implicated in the maintenance of neural stem cells [107], and is crucial for maintenance of muscle stem cell quiescence [108].

### Overlap with other proteomics studies

Other groups have reported serum protein levels that increase with age. However, few are associated with mortality. Findings of three studies [109–111] are shown in Table S4. The proteins of note from these that overlap with our current study, and for which *FOXO3* genotype reduces mortality, include coiled-coil domain-containing protein 80 (CCDC80), EGF-containing fibulin-like extracellular matrix protein 1 (EFEMP1), GDF15, insulin-like growth factor-binding protein 2 (IGFBP2), macrophage metalloelastase (MMP12), SPARC-related modular calcium-binding protein 1 (SMOC1), thrombospondin-2 (THBS2), and WAP four-disulfide core domain protein 2 (WFDC2).

### *FOXO3* resilience genotype

The *FOXO3* longevity genotype is actually a haplotype that includes at least 14 SNPs that have putative functional differences (i.e., transcription factor binding site changes) compared with the more common non-long-lived haplotypes [4]. These SNPs are in a high degree of linkage disequilibrium (LD) (i.e., are closely correlated and co-inherited) in the Japanese population that our subjects belong to. This is because SNP *rs12212067*, while in LD with the others, has a lower minor allele frequency (0.09 vs 0.26) and may reflect a resilience haplotype that overlaps with the entire *FOXO3* longevity haplotype [4]. We reported previously that *FOXO3* SNP *rs12212067* is located close to a promoter for a transcription variant of *FOXO3* that is associated with increased levels of full-length *FOXO3* mRNA isoform in peripheral blood and a decrease in truncated *FOXO3* full-length mRNA isoforms in skeletal muscle RNA [112]. This may suggest that the current resilience genotype is actually distinct, but part of a longevity haplotype. (Please note that herein “isoform” refers to the various mRNA transcripts that can be generated from a gene.)

In support of the significance of this SNP, Lee and colleagues observed a striking genotype-specific difference in the production of cytokines, with PBMC from *rs12212067* minor (*G*) allele homozygotes secreting less TNF-α (which is proinflammatory) than is secreted by major (*T*) allele homozygous individuals [29]. PBMCs from minor allele homozygotes also secreted relatively more IL-10 (anti-inflammatory) in response to higher concentrations of lipopolysaccharide binding protein in PBMCs from healthy donors. They showed that FOXO3 increases inflammatory cytokine levels due to several FOXO3 binding sites in the TGF-β1 promoter. The *G*-allele of *rs12212067* was associated with an increased risk of malaria [113]. Others confirmed that the *G*-allele was associated with an increased inflammatory response to *Plasmodium falciparum* malaria [113]. The finding of increased risk of Crohn’s disease [29, 30] was independently confirmed by Alonso and colleagues [114].

### Limitations

Although 88% of participants were born in Hawaii, there was a theoretical possibility of confounding of *FOXO3* genotypes due to geographic origin.

## Conclusion

The study found 44 “stress” proteins that influence the association of *FOXO3* genotype with reduced mortality. Biological pathways of these proteins suggest that the *FOXO3* resilience genotype functions by reducing mortality by mechanisms relating to innate immunity, BMP signaling, leukocyte migration, and growth factor response.

### Supplementary information

ESM 1 (DOCX 439 kb)

## Data Availability

The datasets generated and analyzed during the current study are available from the corresponding author upon reasonable request.
