# Supplementary material for: Proteomic basis of mortality resilience mediated by FOXO3 longevity genotype
Source: GeroScience. 2023 Mar 7;45(4):2303–24. doi: 10.1007/s11357-023-00740-6 (PMC10651822; doi:10.1007/s11357-023-00740-6)
Supplement: Supplementary file 1 — (DOCX 439 kb) [file 11357_2023_740_MOESM1_ESM.docx]

**Supplement**

**Proteomic basis of mortality resilience mediated by *FOXO3* longevity genotype**

Timothy A. Donlon • Brian J. Morris • Randi Chen • Eunjung Lim • Eric K. Morgen • Kristen Fortney • Naisha Shah • Kamal H Masaki • Bradley J. Willcox

***
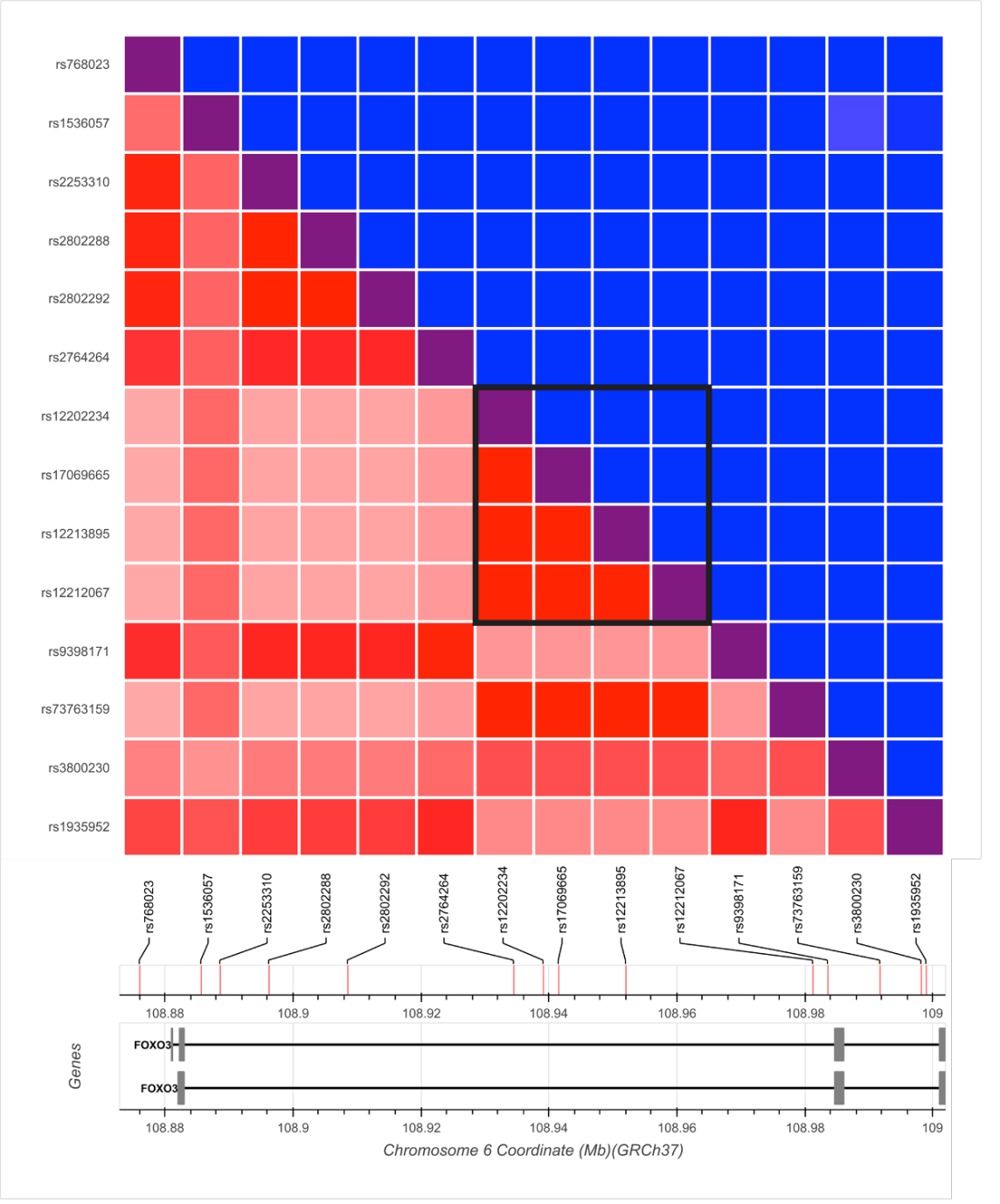
***

**Fig. S1** Location of *FOXO3* resilience variant *rs12212067* relative to 14 other SNP longevity variants in *FOXO3*. The LD matrix plot was constructed using the program LDLink (https://ldlink.nci.nih.gov). The black square denotes the location of the LD block that contains “resilience” variant r*s12212067* and shows that it is not in the highest degree of LD compared to those surrounding it. The most common longevity variant, *rs2802292*, had a minor allele frequency (MAF) of 0.24 while for *rs12212067* MAF was 0.09. The data were from “JPN” population data Phase 3 (Version 5) of the 1000 Genomes Project (<https://www.internationalgenome.org>). Red blocks denote blocks that have a Hedrick’s multiallelic *R^2^* = 1, whereas pink blocks denote blocks that have a *R^2^* value of < 1. Blue blocks denote *D’* values [1].

**
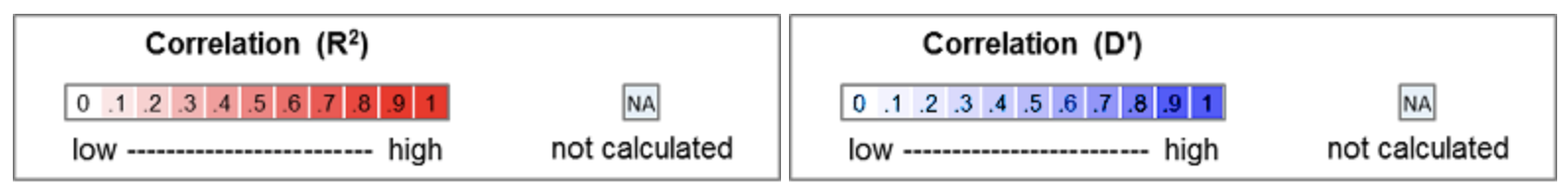
**


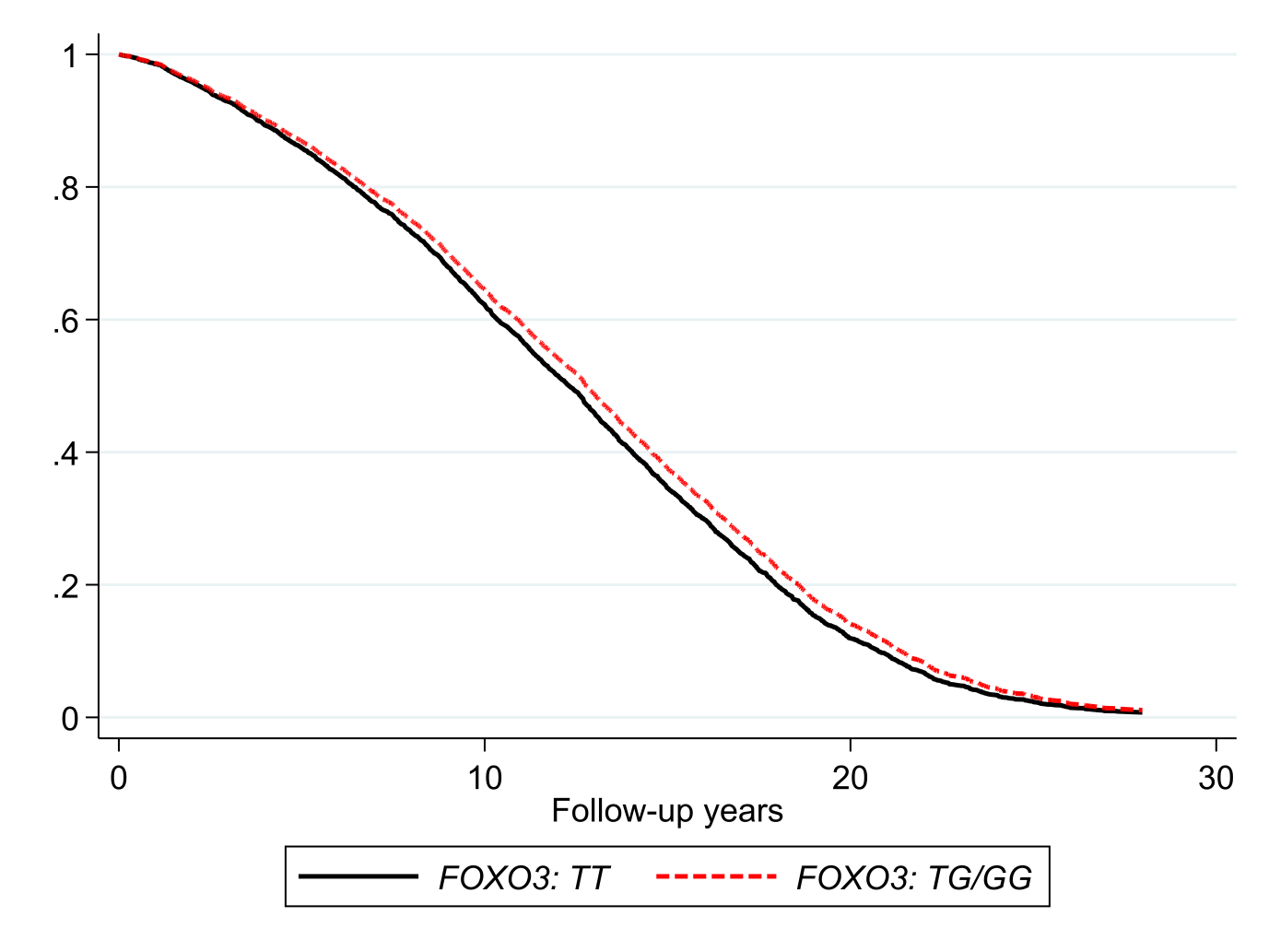


**Fig. S2** Survival curves spanning the period from baseline (1991–1993) to Dec 31, 2019 for carriers of the *FOXO3* longevity-associated (*G*) allele of *FOXO3* SNP *rs12212067*, or who were major allele homozygotes (*TT*). As expected, there was no significant difference between each survival curve (*p*=0.069) because the longevity effect of *FOXO3* genotype only applies to individuals with a cardiometabolic disease [2]. Moreover, our previous study involved the most common longevity variant, *rs2802292*, which has a minor allele frequency (MAF) of 0.24, whereas for *rs12212067* MAF was 0.09. In that study, healthy individuals lived longer and *FOXO3* genotype had no effect on their mortality. The survival probabilities were estimated from the Cox proportional hazard model (see Methods): h(t) = h(t0)*exp(β1*Age + β2*BMI + β3*glucose + β4**FOXO3* (*G*)) by fixing age at 75 years, BMI at the mean, 23.8 kg/m^2^, and glucose at the mean, 111 mg/dL. Ordinate shows proportion still alive.

**Table S1** Baseline (examination 4) characteristics (mean ± SD) by *FOXO3* *rs12212067* genotype.

| **Variables** | ***TT*** | ***TG*/*GG*** | ***p*** |
| --- | --- | --- | --- |
| n | 801 | 174 |  |
| Age (years) | 75.4 ± 2.5 | 75.4 ± 2.5 | 0.65 |
| BMI (kg/m²) | 23.9 ± 3.1 | 23.7 ± 3 | 0.57 |
| Fasting glucose (mg/dl) | 110.9 ± 25.1 | 110.5 ± 23.1 | 0.85 |
| Smoking (pack-years) | 25.9 ± 34.4 | 26 ± 34.5 | 1.00 |
| Alcohol intake (oz/mo) | 19.2 ± 40.3 | 18.3 ± 34.8 | 0.79 |
| Physical activity index | 31.4 ± 4.9 | 31.3 ± 4.5 | 0.96 |

**Table S2** Age-adjusted means of protein concentration for each *FOXO3* genotype shown.

| **Protein/gene name** | **Gene symbol** | **UniProt ID** | ***TT*** (n=801) | ***TG*/*GG* (n=174)** | ***p*** |
| --- | --- | --- | --- | --- | --- |
| Neural EGFL like 2 | *NELL2* | Q99435 | 7.23 ± 0.16 | 7.21 ± 0.15 | 0.33 |
| Growth/differentiation factor 15 | *GDF15* | Q99988 | 10.65 ± 0.35 | 10.62 ± 0.34 | 0.44 |
| Matrilin-2 | *MATN2* | O00339 | 9.25 ± 0.17 | 9.24 ± 0.16 | 0.54 |
| Peptidyl-prolyl cis-trans isomerase C | *PPIC* | P45877 | 9.49 ± 0.2 | 9.48 ± 0.19 | 0.34 |
| EGF-containing fibulin-like extracellular matrix protein 1 | *EFEMP1* | Q12805 | 7.45 ± 0.18 | 7.45 ± 0.19 | 0.84 |
| DnaJ homolog subfamily B member 9 | *DNAJB9* | Q9UBS3 | 9.47 ± 0.24 | 9.45 ± 0.23 | 0.27 |
| WAP four-disulfide core domain protein 2 | *WFDC2* | Q14508 | 8.89 ± 0.27 | 8.87 ± 0.26 | 0.40 |
| Defensin-5 | *DEFA5* | Q01523 | 8.13 ± 0.4 | 8.07 ± 0.45 | 0.091 |
| CD48 antigen | *CD48* | P09326 | 9.4 ± 0.25 | 9.41 ± 0.24 | 0.58 |
| R-spondin-1 | *RSPO1* | Q2MKA7 | 8.42 ± 0.24 | 8.41 ± 0.26 | 0.81 |
| Inter-alpha-trypsin inhibitor heavy chain H3 | *ITIH3* | Q06033 | 7.59 ± 0.3 | 7.58 ± 0.27 | 0.86 |
| Apolipoprotein F | *APOF* | Q13790 | 9.63 ± 0.47 | 9.63 ± 0.49 | 0.85 |
| Triggering receptor expressed on myeloid cells 2 | *TREM2* | Q9NZC2 | 10.07 ± 0.33 | 10.06 ± 0.35 | 0.72 |
| Leucine-rich repeat transmembrane protein | *FLRT2* | O43155 | 9.53 ± 0.26 | 9.52 ± 0.23 | 0.54 |
| Twisted gastrulation protein homolog 1 | *TWSG1* | Q9GZX9 | 7.49 ± 0.14 | 7.49 ± 0.16 | 0.91 |
| Sushi, von Willebrand factor type A, EGF and pentraxin domain-containing protein 1:Sushi 15-18 | *MASP1* | Q4LDE5 | 8.02 ± 0.31 | 8.02 ± 0.35 | 0.90 |
| Tumor necrosis factor receptor superfamily member 11B | *TNFRSF11B* | O00300 | 9.33 ± 0.28 | 9.35 ± 0.29 | 0.34 |
| Kallikrein-11 | *KLK11* | Q9UBX7 | 7.39 ± 0.27 | 7.37 ± 0.22 | 0.43 |
| Brorin | *VWC2* | Q2TAL6 | 9.38 ± 0.25 | 9.35 ± 0.23 | 0.15 |
| Thrombospondin-2 | *THBS2* | P35442 | 9.42 ± 0.44 | 9.45 ± 0.44 | 0.41 |
| SPARC-related modular calcium-binding protein 1 | *SMOC1* | Q9H4F8 | 7.98 ± 0.17 | 7.98 ± 0.25 | 0.72 |
| Ribonuclease 4 | *RNASE4* | P34096 | 9.82 ± 0.18 | 9.81 ± 0.17 | 0.61 |
| Neuroblastoma suppressor of tumorigenicity 1 | *NBL1* | P41271 | 8.01 ± 0.32 | 8 ± 0.33 | 0.65 |
| Slit homolog 2 protein | *SLIT2* | O94813 | 8.97 ± 0.32 | 8.95 ± 0.33 | 0.63 |
| Inhibin beta B chain | *INHBA* | P09529 | 9.7 ± 0.33 | 9.68 ± 0.34 | 0.49 |
| C-X-C motif chemokine 9 | *CXCL9* | Q07325 | 6.57 ± 0.45 | 6.58 ± 0.42 | 0.68 |
| Interleukin-15 receptor subunit alpha | *IL15RA* | Q13261 | 9.64 ± 0.29 | 9.66 ± 0.28 | 0.44 |
| Macrophage metalloelastase | *MMP12* | P39900 | 7.46 ± 0.43 | 7.41 ± 0.43 | 0.14 |
| Elafin/peptidase inhibitor 3 | *PI3* | P19957 | 9.21 ± 0.36 | 9.21 ± 0.41 | 0.8812 |
| Brain-specific serine protease 4 | *PRSS22* | Q9GZN4 | 7.09 ± 0.23 | 7.1 ± 0.21 | 0.63 |
| Collagen alpha-3(VI) chain:bovine pancreatic trypsin inhibitor/Kunitz inhibitor domain, isoform 1 | *COL6A3* | P12111 | 7.91 ± 0.2 | 7.89 ± 0.28 | 0.39 |
| HLA class I histocompatibility antigen, alpha chain G | *HLA-G* | P17693 | 9.69 ± 0.15 | 9.69 ± 0.16 | 0.55 |
| Acylphosphatase-2 | *ACYP2* | P14621 | 9.09 ± 0.27 | 9.1 ± 0.3 | 0.57 |
| Ganglioside GM2 activator | *GM2A* | P17900 | 7.49 ± 0.23 | 7.49 ± 0.26 | 0.96 |
| Cathepsin Z | *CTSZ* | Q9UBR2 | 7.76 ± 0.23 | 7.75 ± 0.22 | 0.74 |
| Insulin-like growth factor-binding protein 2 | *IGFBP2* | P18065 | 10.86 ± 0.46 | 10.87 ± 0.44 | 0.79 |
| Interleukin-18-binding protein | *IL18BP* | O95998 | 8.08 ± 0.24 | 8.08 ± 0.23 | 0.73 |
| Trefoil factor 3 | *TFF3* | Q07654 | 9.08 ± 0.27 | 9.08 ± 0.32 | 0.82 |
| Tumor necrosis factor receptor superfamily member 1B | *TNFRSF1B* | P20333 | 8 ± 0.24 | 8 ± 0.26 | 0.87 |
| Desmocollin-2 | *DSC2* | Q02487 | 7.59 ± 0.21 | 7.59 ± 0.28 | 0.81 |
| Coiled-coil domain-containing protein 80 | *CCDC80* | Q76M96 | 7.95 ± 0.21 | 7.91 ± 0.2 | 0.047 |
| Alpha-parvin | *PARVA* | Q9NVD7 | 7.64 ± 0.22 | 7.64 ± 0.24 | 0.99 |
| Vascular endothelial growth factor D | *VEGFA* | O43915 | 9.81 ± 0.26 | 9.82 ± 0.29 | 0.64 |
| Sushi, von Willebrand factor type A, EGF and pentraxin domain-containing protein 1:EGF-like domains 4-6 | *MASP1* | Q4LDE5 | 7.81 ± 0.33 | 7.81 ± 0.37 | 0.80 |

–––––––––––––––––––––––––––––––––––––––––––––––––––––––––––––––––––––––––––––––––––––––––––––––––––––––

The general linear model (GLM) was used to estimate age-adjusted means of protein concentration for each *FOXO3* genotype.

In general, the *FOXO3* genotype was not associated with “stress protein” concentration. The exception was CCDC80, which showed a significant association with *FOXO3* genotype, but became nonsignificant after correction for multiple testing. Apparently, the resilience effect of *FOXO3* genotype on mortality may not be by directly modifying or changing the concentration of stress factor (protein), but by other mechanisms (see Discussion in main manuscript).

**Table S3** Functions* of the top 35 stress proteins influenced by *FOXO3* genotype leading to reduced mortality.

| **Protein/gene name** | **Gene symbol** | **Definition and function** | **Pathway** |
| --- | --- | --- | --- |
| Neural EGFL like 2 | *NELL2* | A glycoprotein containing several von Willebrand factor C domains and epidermal growth factor (EGF)-like domains. The encoded protein acts as a homotrimer and is found in the cytoplasm. Several variants encoding several different isoforms exist, and at least one isoform appears to be a secreted protein. Studies in mouse suggest that this protein plays a role in neural cell growth and differentiation as well as in oncogenesis. Required for neuron survival through the modulation of MAPK pathways (by similarity). Involved in the regulation of hypothalamic GNRH secretion and the control of puberty (by similarity). | Signal transduction/growth |
| Growth/differentiation factor 15 | *GDF15* | A secreted ligand of the TGF-beta (transforming growth factor-beta) superfamily of proteins. Ligands of this family bind various TGF-beta receptors leading to recruitment and activation of SMAD family transcription factors that regulate gene expression. The encoded preproprotein is proteolytically processed to generate each subunit of the disulfide-linked homodimer. The protein is expressed in a broad range of cell types, acts as a pleiotropic cytokine and is involved in the stress response program of cells after cellular injury. Increased protein levels are associated with disease states such as tissue hypoxia, inflammation, acute injury and oxidative stress. Diseases associated with GDF15 include hereditary transthyretin amyloidosis and myalgic encephalomyelitis/chronic fatigue syndrome. Among its related pathways are CREB pathway and ERK signaling. Gene Ontology (GO) annotations related to this gene include cytokine activity and transforming growth factor beta receptor binding. An important paralog of this gene is INHBE. GDF15 regulates food intake, energy expenditure and body weight in response to metabolic and toxin-induced stresses. Binds to its receptor, GFRAL, and activates GFRAL-expressing neurons localized in the area postrema and nucleus tractus solitarius of the brainstem. It then triggers the activation of neurons localized within the parabrachial nucleus and central amygdala, which contsitutes part of the 'emergency circuit' that shapes feeding responses to stressful conditions. On hepatocytes, GDF15 inhibits growth hormone signaling. | Signal transduction/growth |
| Matrilin-2 | *MATN2* | A member of the von Willebrand factor A domain containing protein family. This family of proteins is thought to be involved in the formation of filamentous networks in the extracellular matrices of various tissues. It contains five von Willebrand factor A domains. The specific function has not yet been determined. Diseases associated with MATN2 include pilocytic astrocytoma. GO annotations related to MATN2 include calcium ion binding. Is involved in matrix assembly. | Extracellular matrix |
| Peptidyl-prolyl cis-trans isomerase C | *PPIC* | Is a member of the peptidyl-prolyl cis-trans isomerase (PPIase)) family. PPIases catalyze the cis-trans isomerization of proline imidic peptide bonds in oligopeptides and accelerate the folding of proteins. Similar to other PPIases, PPIC can bind immunosuppressant cyclosporin A. | Chaperone |
| EGF-containing fibulin-like extracellular matrix protein 1 | *EFEMP1* | A member of the fibulin family of extracellular matrix glycoproteins. Like all members of this family, the encoded protein contains tandemly repeated epidermal growth factor-like repeats followed by a C-terminus fibulin-type domain. Its gene is upregulated in malignant gliomas and may play a role in the aggressive nature of these tumors. Mutations in its gene are associated with Doyne honeycomb retinal dystrophy. Among its related pathways are integrin pathway and ERK signaling. GO annotations related to EFEMP1 include calcium ion binding and epidermal growth factor receptor binding. Binds EGFR, the EGF receptor, inducing EGFR autophosphorylation and the activation of downstream signaling pathways. May play a role in cell adhesion and migration. May function as a negative regulator of chondrocyte differentiation. In the olfactory epithelium, it may regulate glial cell migration, differentiation and the ability of glial cells to support neuronal neurite outgrowth. | Extracellular matrix |
| DnaJ homolog subfamily B member 9 | *DNAJB9* | J proteins function in many cellular processes by regulating the ATPase activity of 70 kDa heat shock proteins. DNAJB9 is a member of the type 2 subgroup of DnaJ proteins. Is localized to the endoplasmic reticulum. Is induced by endoplasmic reticulum stress and plays a role in protecting stressed cells from apoptosis. Diseases associated with DNAJB9 include fibrillary glomerulonephritis and non-gestational choriocarcinoma. Among its related pathways are metabolism of proteins and unfolded protein response (UPR). Gene Ontology annotations related to this protein include misfolded protein binding. Co-chaperone for Hsp70 protein HSPA5/BiP that acts as a key repressor of the ERN1/IRE1-mediated unfolded protein response (UPR) (by similarity). J domain-containing co-chaperones stimulate the ATPase activity of Hsp70 proteins and are required for efficient substrate recognition by Hsp70 proteins [3]. In the unstressed endoplasmic reticulum, it interacts with the luminal region of ERN1/IRE1 and selectively recruits HSPA5/BiP. HSPA5/BiP disrupts the dimerization of the active ERN1/IRE1 luminal region, thereby inactivating ERN1/IRE1 (by similarity). Also involved in endoplasmic reticulum-associated degradation (ERAD) of misfolded proteins. Required for survival of B-cell progenitors and normal antibody production (by similarity). | Chaperone |
| WAP four-disulfide core domain protein 2 | *WFDC2* | A member of the WFDC domain family. The WFDC domain, or WAP signature motif, contains eight cysteines forming four disulfide bonds at the core of the protein, and functions as a protease inhibitor in many family members. Its gene is expressed in pulmonary epithelial cells, and in some ovarian cancers. WFDC2 is a small secretory protein that may be involved in sperm maturation. | Unclear |
| Defensin-5 | *DEFA5* | Defensins are a family of antimicrobial and cytotoxic peptides thought to be involved in host defense. They are abundant in the granules of neutrophils and also found in the epithelia of mucosal surfaces such as those of the intestine, respiratory tract, urinary tract, and vagina. Members of the defensin family are highly similar in protein sequence and distinguished by a conserved cysteine motif. Several of the alpha defensin genes appear to be clustered on chromosome 8. The protein encoded by this gene, defensin, alpha 5, is highly expressed in the secretory granules of Paneth cells of the ileum. Diseases associated with DEFA5 include Crohn's colitis and urethritis. Among its related pathways are defensins and innate immune system. Has antimicrobial activity against Gram-negative and Gram-positive bacteria. Defensins are thought to kill microbes by permeabilizing their plasma membrane. All DEFA5 peptides exert antimicrobial activities, but their potency is affected by peptide processing. | Antimicrobial |
| CD48 antigen | *CD48* | CD48 is a neutrophil and pancreatic elastase-specific inhibitor of skin. It may prevent elastase-mediated tissue proteolysis. Has been shown to inhibit the alpha-4-beta-2/CHRNA2-CHRNB2 nicotinic acetylcholine receptor and to produce a weak inhibition on Kv11.1/KCNH2/ERG1 and on the transient receptor potential cation channel subfamily V member 1 (TRPV1) | Extracellular matrix |
| R-spondin-1 | *RSPO1* | Is a secreted activator protein with two cysteine-rich, furin-like domains and one thrombospondin type 1 domain. The encoded protein is a ligand for leucine-rich repeat-containing G-protein coupled receptors (LGR proteins) and positively regulates the Wnt signaling pathway. In mice, the protein induces the rapid onset of crypt cell proliferation and increases intestinal epithelial healing, providing a protective effect against chemotherapy-induced adverse effects. Diseases associated with RSPO1 include palmoplantar hyperkeratosis with squamous cell carcinoma of skin and 46,XX sex reversal and hermaphroditism. Among its related pathways are Wnt signaling network and signaling by GPCR. Gene Ontology annotations related to this protein include signaling receptor binding and G protein-coupled receptor binding. | Signal transduction/growth |
| Inter-alpha-trypsin inhibitor heavy chain H3 | *ITIH3* | Inter-Alpha-Trypsin Inhibitor Heavy Chain 3 (ITIH3) is the heavy chain subunit of the pre-alpha-trypsin inhibitor complex. This complex may stabilize the extracellular matrix through its ability to bind hyaluronic acid. The ITIH3 gene is present in an inter-alpha-trypsin inhibitor family gene cluster on chromosome 3. Gene polymorphisms may be associated with increased risk for schizophrenia and major depressive disorder. Among its related pathways are response to elevated platelet cytosolic Ca^2+^ and cell adhesion cell-matrix glycoconjugates. Gene Ontology annotations include serine-type endopeptidase inhibitor activity and endopeptidase inhibitor activity. An important paralog is ITIH1. May act as a carrier of hyaluronan in serum or as a binding protein between hyaluronan and other matrix protein, including those on cell surfaces in tissues to regulate the localization, synthesis and degradation of hyaluronan which are essential to cells undergoing biological processes. | Extracellular matrix |
| Apolipoprotein F | *APOF* | Is one of the minor apolipoproteins found in plasma. Forms complexes with lipoproteins and may be involved in transport and/or esterification of cholesterol. Among its related pathways are lipoprotein metabolism and metabolism. Gene Ontology annotations include signaling receptor binding and lipid transporter activity. Is a minor apolipoprotein that associates with LDL. Inhibits cholesteryl ester transfer protein (CETP) activity and appears to be an important regulator of cholesterol transport. Also associates to a lesser degree with VLDL, Apo-AI and Apo-AII. | Lipid metabolism |
| Triggering receptor expressed on myeloid cells 2 | *TREM2* | Membrane protein that forms a receptor signaling complex with the TYRO protein tyrosine kinase binding protein. Functions in immune response and may be involved in chronic inflammation by triggering the production of constitutive inflammatory cytokines. Genetic defects are a cause of polycystic lipomembranous osteodysplasia with sclerosing leukoencephalopathy (PLOSL) 1 and 2. Among its related pathways are neuroscience and immunoregulatory interactions between a lymphoid and a non-lymphoid cell. Gene Ontology annotations related to TREM2 include signaling receptor activity and lipopolysaccharide binding. An important paralog is CD300A. Forms a receptor signaling complex with TYROBP which mediates signaling and cell activation following ligand binding [4]. Acts as a receptor for amyloid-beta protein 42, a cleavage product of the amyloid-beta precursor protein APP, and mediates its uptake and degradation by microglia [5,6]. Binding to amyloid-beta 42 mediates microglial activation, proliferation, migration, apoptosis and expression of pro-inflammatory cytokines, such as IL6R and CCL3, and the anti-inflammatory cytokine ARG1 (by similarity). Acts as a receptor for lipoprotein particles such as LDL, VLDL, and HDL and for apolipoproteins such as APOA1, APOA2, APOB, APOE, APOE2, APOE3, APOE4, and CLU and enhances their uptake in microglia [5]. TREM2 binds phospholipids (preferably anionic lipids) such as phosphatidylserine, phosphatidylethanolamine, phosphatidylglycerol and sphingomyelin [7]. Regulates microglial proliferation by acting as an upstream regulator of the Wnt/beta-catenin signaling cascade (by similarity). Required for microglial phagocytosis of apoptotic neurons [8]. Is also required for microglial activation and phagocytosis of myelin debris after neuronal injury and of neuronal synapses during synapse elimination in the developing brain (by similarity). Regulates microglial chemotaxis and process outgrowth, and also the microglial response to oxidative stress and lipopolysaccharide (by similarity). It suppresses PI3K and NF-kappa-B signaling in response to lipopolysaccharide, thus promoting phagocytosis, suppressing pro-inflammatory cytokine and nitric oxide production, inhibiting apoptosis and increasing expression of IL10 and TGF-beta (by similarity). During oxidative stress, it promotes anti-apoptotic NF-kappa-B signaling and ERK signaling (by similarity). Plays a role in microglial mTOR activation and metabolism (by similarity). Regulates age-related changes in microglial numbers [9]. Triggers activation of the immune responses in macrophages and dendritic cells [4]. Mediates cytokine-induced formation of multinucleated giant cells which are formed by the fusion of macrophages (by similarity). In dendritic cells, TREM3 mediates up-regulation of chemokine receptor CCR7 and dendritic cell maturation and survival [10]. Involved in the positive regulation of osteoclast differentiation. | Signal transduction/growth |
| Leucine-rich repeat transmembrane protein FLRT2 | *FLRT2* | Fibronectin leucine rich transmembrane protein 2 (FLRT2) is a member of the fibronectin leucine rich transmembrane (FLRT) family of cell adhesion molecules, which regulate early embryonic vascular and neural development. Is a type I transmembrane protein which has an extracellular region consisting of an N-terminal leucine-rich repeat domain and a type 3 fibronectin domain, followed by a transmembrane domain and a short C-terminal cytoplasmic tail domain. It functions as both a homophilic cell adhesion molecule and a heterophilic chemorepellent through its interaction with members of the uncoordinated-5 receptor family. Proteolytic removal of the extracellular region controls the migration of neurons in the developing cortex. Among its related pathways are downstream signaling of activated FGFR2 and signaling by GPCR. Gene Ontology annotations related to FLRT2 include protein-macromolecule adaptor activity and chemorepellent activity. An important paralog is FLRT3. Functions in cell-cell adhesion, cell migration and axon guidance. Mediates cell-cell adhesion via its interactions with ADGRL3 and probably also other iatrophilins that are expressed at the surface of adjacent cells. May play a role in the migration of cortical neurons during brain development via its interaction with UNC5D. Mediates axon growth cone collapse and plays a repulsive role in neuron guidance via its interaction with UNC5D, and possibly also other UNC-5 family members. Plays a role in fibroblast growth factor-mediated signaling cascades. Required for normal organization of the cardiac basement membrane during embryogenesis, and for normal embryonic epicardium and heart morphogenesis. | Extracellular matrix |
| Twisted gastrulation protein homolog 1 | *TWSG1* | Enables transforming growth factor beta binding activity. Involved in several processes, including negative regulation of CD4-positive, alpha-beta T cell proliferation. Positive regulation of pathway-restricted SMAD protein phosphorylation, and transforming growth factor beta receptor signaling pathway. Predicted to be located in extracellular region. May be involved in dorsoventral axis formation. Seems to antagonize BMP signaling by forming ternary complexes with CHRD and BMPs, thereby preventing BMPs from binding to their receptors. In addition to the anti-BMP function, it also has pro-BMP activity, partly mediated by cleavage and degradation of CHRD, which releases BMPs from ternary complexes. May be an important modulator of BMP-regulated cartilage development and chondrocyte differentiation. May play a role in thymocyte development (by similarity). | Signal transduction/growth |
| Sushi, von Willebrand factor type A, EGF and pentraxin domain-containing protein 1 | *MASP1* | SVEP1 is a serine protease that functions as a component of the lectin pathway of complement activation. The complement pathway plays an essential role in the innate and adaptive immune response. Is synthesized as a zymogen and is activated when it complexes with the pathogen recognition molecules of lectin pathway, the mannose-binding lectin and the ficolins. This protein is not directly involved in complement activation but may play a role as an amplifier of complement activation by cleaving complement C2 or by activating another complement serine protease, SVEP1. SVEP1 is also able to cleave fibrinogen and factor XIII and may be involved in coagulation. A splice variant which lacks the serine protease domain functions as an inhibitor of the complement pathway. Diseases associated with SVEP1 include 3MC syndrome 1 and 3MC syndrome, which encompass 4 rare autosomal recessive disorders that were previously designated the Carnevale, Mingarelli, Malpuech, and Michel’s syndromes. Among its related pathways are binding and uptake of ligands by scavenger receptors and immune response Lectin induced complement pathway. Gene Ontology annotations related to MASP1include calcium ion binding and serine-type endopeptidase activity. An important paralog of its gene is *MASP2*. Functions in the lectin pathway of complement, which performs a key role in innate immunity by recognizing pathogens through patterns of sugar moieties and neutralizing them. The lectin pathway is triggered upon binding of mannan-binding lectin (MBL) and ficolins to sugar moieties which leads to activation of the associated proteases MASP1 and MASP2. Functions as an endopeptidase and may activate MASP2 or C2 or directly activate C3, the key component of complement reaction. Isoform 2 may have an inhibitory effect on the activation of the lectin pathway of complement or may cleave IGFBP5. Also plays a role in development. | Innate immunity |
| Tumor necrosis factor receptor superfamily member 11B | *TNFRSF11B* | Is a member of the TNF-receptor superfamily. TNFRSF11B is an osteoblast-secreted decoy receptor that functions as a negative regulator of bone resorption. It specifically binds to its ligand, osteoprotegerin ligand, both of which are key extracellular regulators of osteoclast development. Studies of the mouse counterpart suggest that this protein and its ligand play a role in lymph-node organogenesis and vascular calcification. Diseases associated with TNFRSF11B include Paget disease of bone 5, juvenile-onset and bone disease. Among its related pathways are cytokine signaling in immune system and osteoblast signaling. Gene Ontology annotations related to TNFRSF11B include signaling receptor activity and cytokine activity. | Signal transduction/growth |
| Kallikrein-11 | *KLK11* | Kallikrein-11 (KLK11) is a member of the kallikrein gene family. Kallikreins are a subgroup of serine proteases having diverse physiological functions. Growing evidence suggests that many kallikreins are implicated in carcinogenesis and some have potential as novel cancer and other disease biomarkers. KLK11 is one of the 15 kallikrein subfamily members located in a cluster on chromosome 19. Alternate splicing and the use of alternate promoters results in multiple transcript variants encoding distinct isoforms which are differentially expressed. Diseases associated with KLK11 include prostate cancer. Among its related pathways are collagen formation and MSP-RON signaling. Gene Ontology annotations related KLK11 include serine-type endopeptidase activity and serine-type peptidase activity. An important paralog is KLK5. | Signal transduction/growth |
| Brorin | *VWC2* | Brorin (alternative names: Von Willebrand factor C domain containing 2 (VWC2) and brain-specific cordin-like protein) is a secreted bone morphogenic protein antagonist. Brorin is possibly involved in neural function and development and may have a role in cell adhesion. Diseases associated with brorin include spinocerebellar ataxia 36. An important paralog of its gene is *VWC2*. Is a BMP antagonist that may have a role in neural development. Promotes cell adhesion (by similarity). Primary accession: Q2TAL6. | Extracellular matrix |
| Thrombospondin-2 | *THBS2* | Thrombospondin-2 is a member of the thrombospondin family. It is a disulfide-linked homotrimeric glycoprotein that mediates cell-to-cell and cell-to-matrix interactions. This protein has been shown to function as a potent inhibitor of tumor growth and angiogenesis. Studies of the mouse counterpart suggest that it may modulate the cell surface properties of mesenchymal cells and be involved in cell adhesion and migration. Diseases associated with it include intervertebral disc disease and back pain. Among its related pathways are HIV life cycle and O-glycosylation of TSR domain-containing proteins. Gene Ontology annotations related to thrombospondin-2 include calcium ion binding and heparin binding. | Extracellular matrix |
| SPARC-related modular calcium-binding protein 1 | *SMOC1* | Is a multi-domain secreted protein that may have a critical role in ocular and limb development. Mutations in the SMOC1 gene are associated with microphthalmia and limb anomalies. Alternatively-spliced transcript variants encoding different isoforms have been found. Diseases associated with SMOC1 include microphthalmia with limb anomalies and microphthalmia. Gene Ontology annotations related to SMOC1 include calcium ion binding and extracellular matrix binding. Plays essential roles in both eye and limb development. Probable regulator of osteoblast differentiation. | Extracellular matrix |
| Ribonuclease 4 | *RNASE4* | Ribonuclease 4 belongs to the pancreatic ribonuclease family. It plays an important role in mRNA cleavage and has marked specificity towards the 3' side of uridine nucleotides. Alternative splicing results in four transcript variants encoding the same protein. Its gene and the gene that encodes angiogenin share promoters and 5' exons. Each gene splices to a unique downstream exon that contains its complete coding region. Diseases associated with the RNASE4 gene include amyotrophic lateral sclerosis 9. Gene Ontology annotations related to ribonuclease 4include nucleic acid binding and ribonuclease activity. | RNA processing |
| UV excision repair protein RAD23 homolog B | *RAD23B* | RAD23B is one of two human homologs of *Saccharomyces cerevisiae* Rad23, a protein involved in the nucleotide excision repair (NER). Is a component of the protein complex that specifically complements the NER defect of xeroderma pigmentosum group C (XP-c) cell extracts in vitro. Interacts with, and elevates, the nucleotide excision activity of 3-methyladenine-DNA glycosylase (MPG), which suggested a role in DNA damage recognition in base excision repair. RAD23B contains an N-terminal ubiquitin-like domain, which was reported to interact with 26S proteasome, and thus this protein may be involved in the ubiquitin mediated proteolytic pathway in cells. Alternative splicing results in multiple transcript variants encoding distinct isoforms. Diseases associated with RAD23B include xeroderma pigmentosum, complementation group C and xeroderma pigmentosum variant type. Among its related pathways are DNA repair pathways, full network and calnexin/calreticulin cycle. Gene Ontology annotations related to *RAD23B* include single-stranded DNA binding and polyubiquitin modification-dependent protein binding. An important paralog of *RAD23B* is RAD23A. Is a multiubiquitin chain receptor involved in modulation of proteasomal degradation. Binds to polyubiquitin chains. Proposed to be capable to bind simultaneously to the 26S proteasome and to polyubiquitinated substrates and to deliver ubiquitinated proteins to the proteasome. May play a role in endoplasmic reticulum-associated degradation (ERAD) of misfolded glycoproteins by association with PNGase and delivering deglycosylated proteins to the proteasome. Is involved in global genome nucleotide excision repair (GG-NER) by acting as component of the XPC complex. Cooperatively with CETN2 it appears to stabilize XPC. May protect XPC from proteasomal degradation. | Autophagy |
| Neuroblastoma suppressor of tumorigenicity 1 | *NBL1* | NBL1 is the founding member of the evolutionarily conserved cerberus and Dan (can) family of proteins, which contain a domain resembling the C-terminal cystine knot-like (CTCK) motif found in a number of signaling molecules. These proteins are secreted, and act as bone morphogenetic protein (BMP) antagonists by binding to BMPs and preventing them from interacting with their receptors. They may thus play an important role during growth and development. Alternatively-spliced transcript variants have been identified for the NBL1 gene. Read-through transcripts between this locus and the upstream mitochondrial inner membrane organizing system 10 gene (*MICOS10*) have been observed. Diseases associated with NBL1 include neuroblastoma and nodular ganglioneuroblastoma. Gene Ontology annotations related to *NBL1* include protein homodimerization activity and morphogen activity. | Signal transduction/growth |
| Slit homolog 2 protein | *SLIT2* | Slit homolog 2 protein is a member of the slit family of secreted glycoproteins, which are ligands for the Robo family of immunoglobulin receptors. Slit proteins play highly conserved roles in axon guidance and neuronal migration and may also have functions during other cell migration processes including leukocyte migration. Diseases associated with SLIT2 include Cakut and Crohn's colitis. Among its related pathways are guidance cues and growth cone motility and signaling by robo receptor. Gene Ontology annotations related to SLIT2 include calcium ion binding and identical protein binding. | Extracellular matrix |
| Inhibin beta B chain | *INHBA* | Inhibin beta B chain is a member of the transforming growth factor-beta (TGF-beta) superfamily of proteins. Its preproprotein is proteolytically processed to generate a subunit of the dimeric activin and inhibin protein complexes. These complexes activate and inhibit, respectively, follicle stimulating hormone secretion from the pituitary gland. The protein also plays a role in eye, tooth and testis development. Elevated expression of the INHBA gene may be associated with cancer cachexia in human patients. Diseases associated with INHBA include ovary adenocarcinoma and sex cord-gonadal stromal tumor. Among its related pathways are embryonic and induced pluripotent stem cells and lineage-specific markers and cardiac progenitor differentiation. Gene Ontology annotations related to INHBA include identical protein binding and signaling receptor binding. An important paralog is INHBB. Inhibins and activins inhibit and activate, respectively, the secretion of follitropin by the pituitary gland. Inhibins/activins are involved in regulating a number of diverse functions such as hypothalamic and pituitary hormone secretion, gonadal hormone secretion, germ cell development and maturation, erythroid differentiation, insulin secretion, nerve cell survival, embryonic axial development or bone growth, depending on their subunit composition. Inhibins appear to oppose the functions of activins. | Signal transduction/growth |
| C-X-C motif chemokine 9 | *CXCL9* | CXCL9 is an antimicrobial protein that is part of a chemokine superfamily of secreted proteins involved in immunoregulatory and inflammatory processes. CXCL9 is thought to be involved in T cell trafficking. CXCL9 binds to C-X-C motif chemokine 3 and is a chemoattractant for lymphocytes but not for neutrophils. Diseases associated with CXCL9 include proliferative glomerulonephritis and paracoccidioidomycosis. Among its related pathways are PEDF induced signaling and peptide ligand-binding receptors. Gene Ontology annotations related to CXCL9 include cytokine activity and CXCR3 chemokine receptor binding. An important paralog is CXCL10. Is a cytokine that affects the growth, movement, or activation state of cells that participate in immune and inflammatory response. Is chemotactic for activated T-cells. Binds to CXCR3. | Innate immunity |
| Interleukin-15 receptor subunit alpha | *IL15RA* | IL15R-alpha is a cytokine receptor that specifically binds interleukin-15 (IL-15) with high affinity. The receptors of IL-15 and IL-2 share two subunits, IL2R-beta and IL2R-gamma. This forms the basis of many overlapping biological activities of IL-15 and IL-2. IL15R-alpha is structurally related to IL2R-alpha, an additional IL-2-specific alpha subunit necessary for high affinity IL-2 binding. Unlike IL2R-alpha, IL15R-alpha is capable of binding IL-15 with high affinity independent of other subunits, which suggests distinct roles between IL-15 and IL-2. This receptor is reported to enhance cell proliferation and expression of apoptosis inhibitor BCL2L1/BCL2-XL and BCL2. Diseases associated with the IL15RA gene include T-cell large granular lymphocyte leukemia and ossification of the posterior longitudinal ligament of spine. Among its related pathways are cytokine signaling in immune system and PEDF induced signaling. Gene Ontology annotations include obsolete signal transducer activity and cytokine receptor activity. | Innate immunity |
| Macrophage metalloelastase | *MMP12* | Macrophage metalloelastase is member of the peptidase M10 family of matrix metalloproteinases (MMPs). Proteins in this family are involved in the breakdown of extracellular matrix in normal physiological processes, such as embryonic development, reproduction, and tissue remodeling, as well as in disease processes, such as arthritis and metastasis. The encoded preproprotein is proteolytically processed to generate the mature protease. This protease degrades soluble and insoluble elastin. Macrophage metalloelastase may play a role in aneurysm formation. Mutations in *MMP12* are associated with lung function and chronic obstructive pulmonary disease (COPD). May be involved in tissue injury and remodeling. Has significant elastolytic activity. Can accept large and small amino acids at the P1' site but has a preference for leucine. Aromatic or hydrophobic residues are preferred at the P1 site, with small hydrophobic residues (preferably alanine) occupying P3. | Extracellular matrix |
| Elafin (peptidase inhibitor 3) | *PI3* | Elafin/peptidase inhibitor 3 is an elastase-specific inhibitor that functions as an antimicrobial peptide against Gram-positive and Gram-negative bacteria, and fungal pathogens. The protein contains a WAP-type four-disulfide core (WFDC) domain and is thus a member of the WFDC domain family. Most WFDC gene members are localized to chromosome 20q12-q13 in two clusters: centromeric and telomeric. This gene belongs to the centromeric cluster. Expression of *PI3* is upregulated by bacterial lipopolysaccharides and cytokines. Diseases associated with PI3 include pustular psoriasis and impetigo herpetiformis. Among its related pathways are defensins and innate immune system. Gene Ontology annotations include serine-type endopeptidase inhibitor activity and peptidase inhibitor activity. An important paralog is SLPI. | Antimicrobial |
| Brain-specific serine protease 4 | *PRSS22* | This gene encodes a member of the trypsin family of serine proteases. The enzyme is expressed in the airways in a developmentally regulated manner. The gene is part of a cluster of serine protease genes on chromosome 16. Gene Ontology (GO) annotations related to this gene include serine-type endopeptidase activity. | Unclear |
| Collagen alpha-3 (VI) chain | *COL6A3* | The alpha-3 chain is one of the three alpha chains of type VI collagen, a beaded filament collagen found in most connective tissues. The alpha-3 chain of type VI collagen is much larger than the alpha-1 and -2 chains. This difference in size is largely due to an increase in the number of subdomains, similar to von Willebrand factor type A domains that are found in the amino terminal globular domain of all the alpha chains. These domains have been shown to bind extracellular matrix proteins, an interaction that explains the importance of this collagen in organizing matrix components. Mutations in the type VI collagen genes are associated with Bethlem myopathy, a rare autosomal dominant proximal myopathy with early childhood onset. Mutations in *COL6A3* are also a cause of Ullrich congenital muscular dystrophy, also referred to as Ullrich scleroatonic muscular dystrophy, an autosomal recessive congenital myopathy that is more severe than Bethlem myopathy. Multiple transcript variants have been identified, but the full-length nature of only some of these variants has been described. | Extracellular matrix |
| HLA class I histocompatibility antigen, alpha chain G | *HLA-G* | HLA class I histocompatibility antigen, alpha chain G (HLA-G) belongs to the HLA class I heavy chain paralogs. Diseases associated with HLA-G include asthma and severe pre-eclampsia. Among its related pathways are antigen presentation-folding, assembly and peptide loading of class I MHC and class I MHC-mediated antigen processing and presentation. Gene Ontology annotations related to HLA-G include protein homodimerization activity and peptide antigen binding.  Isoform 1: Non-classical major histocompatibility class Ib molecule involved in immune regulatory processes at the maternal-fetal interface [11-13]. In complex with B2M/beta-2 microglobulin it binds a limited repertoire of nonamer self-peptides derived from intracellular proteins including histones and ribosomal proteins [14,15]. Peptide-bound HLA-G-B2M complex acts as a ligand for inhibitory/activating KIR2DL4, LILRB1 and LILRB2 receptors on uterine immune cells to promote fetal development while maintaining maternal-fetal tolerance [11-13,16-18]. Upon interaction with KIR2DL4 and LILRB1 receptors on decidual NK cells, it triggers NK cell senescence-associated secretory phenotype as a molecular switch to promote vascular remodeling and fetal growth in early pregnancy [11-13,16]. Through interaction with KIR2DL4 receptor on decidual macrophages induces proinflammatory cytokine production mainly associated with tissue remodeling [13]. Through interaction with LILRB2 receptor triggers differentiation of type 1 regulatory T cells and myeloid-derived suppressor cells, both of which actively maintain maternal-fetal tolerance [17,18]. May play a role in balancing tolerance and antiviral-immunity at maternal-fetal interface by keeping in check the effector functions of NK, CD8+ T cells and B cells [19-21]. Reprograms B cells toward an immune suppressive phenotype via LILRB1 [21]. May induce immune activation/suppression via intercellular membrane transfer (trogocytosis), likely enabling interaction with KIR2DL4, which resides mostly in endosomes [22,23]. Through interaction with the inhibitory receptor CD160 on endothelial cells may control angiogenesis in immune privileged sites [24].  Isoform 2: Likely does not bind B2M and presents peptides. Negatively regulates NK cell- and CD8+ T cell-mediated cytotoxicity [20].  Isoform 3: Likely does not bind B2M and presents peptides. Negatively regulates NK cell- and CD8+ T cell-mediated cytotoxicity [20].  Isoform 4: Likely does not bind B2M and presents peptides. Negatively regulates NK cell- and CD8+ T cell-mediated cytotoxicity [20].  Isoform 5: Non-classical major histocompatibility class Ib molecule involved in immune regulatory processes at the maternal-fetal interface [11-13]. In complex with B2M/beta-2 microglobulin binds a limited repertoire of nonamer self-peptides derived from intracellular proteins including histones and ribosomal proteins [14,15]. Peptide-bound HLA-G-B2M complex acts as a ligand for inhibitory/activating KIR2DL4, LILRB1 and LILRB2 receptors on uterine immune cells to promote fetal development while maintaining maternal-fetal tolerance [11-13,16,17]. Upon interaction with KIR2DL4 and LILRB1 receptors on decidual NK cells, it triggers NK cell senescence-associated secretory phenotype as a molecular switch to promote vascular remodeling and fetal growth in early pregnancy [11-13,16]. Through interaction with KIR2DL4 receptor on decidual macrophages induces proinflammatory cytokine production mainly associated with tissue remodeling [13]. Through interaction with LILRB2 receptor triggers differentiation of type 1 regulatory T cells and myeloid-derived suppressor cells, both of which actively maintain maternal-fetal tolerance [17]. Reprograms B cells toward an immune suppressive phenotype via LILRB1 [21]. Isoform 6: Likely does not bind B2M and presents peptides. Isoform 7: Likely does not bind B2M and presents peptides. | immune |
| Trefoil factor 3 | *TFF3* | Members of the trefoil family are characterized by having at least one copy of the trefoil motif, a 40-amino acid domain that contains three conserved disulfides. They are stable secretory proteins expressed in gastrointestinal mucosa. Their functions are not defined, but they may protect the mucosa from insults, stabilize the mucus layer and affect healing of the epithelium. Trefoil factor 3 is expressed in goblet cells of the intestines and colon. The gene *TFF3* and two other related trefoil family member genes are found in a cluster on chromosome 21. Involved in the maintenance and repair of the intestinal mucosa. Promotes the mobility of epithelial cells in healing processes (motogen). | growth |
| Tumor necrosis factor receptor superfamily member 1B | *TNFRSF1B* | TNFRSF1B and TNF-receptor 1 form a heterocomplex that mediates the recruitment of two anti-apoptotic proteins, c-IAP1 and c-IAP2, that possess E3 ubiquitin ligase activity. The function of IAPs in TNF-receptor signaling is unknown. However, c-IAP1 is thought to potentiate TNF-induced apoptosis by the ubiquitination and degradation of TNF-receptor-associated factor 2, which mediates anti-apoptotic signals. Knockout studies in mice also suggest a role of this protein in protecting neurons from apoptosis by stimulating antioxidative pathways. Diseases associated with TNFRSF1B include mycosis fungoides and Sezary's disease. Among its related pathways are cytokine signaling in immune system and apoptotic pathways triggered by HIV1. Gene Ontology annotations related to TNFRSF1B include ubiquitin protein ligase binding and tumor necrosis factor-activated receptor activity. TNFRSF1B is a receptor with high affinity for TNFSF2/TNF-alpha and approximately 5-fold lower affinity for homotrimeric TNFSF1/lymphotoxin-alpha. The TRAF1/TRAF2 complex recruits the apoptotic suppressors BIRC2 and BIRC3 to TNFRSF1B/TNFR2. This receptor mediates most of the metabolic effects of TNF-alpha. Isoform 2 blocks TNF-alpha-induced apoptosis, which suggests that it regulates TNF-alpha function by antagonizing its biological activity. | signal transduction/growth |

The information on gene function was taken largely from GeneCards [25].

**Table S4** Validation of the present (top 20) results with previous studies.

|  | Study  _________________________________ | | |
| --- | --- | --- | --- |
| Gene/Protein | **1** | **2** | **3** |
| __________ | __________ | ___________ | ___________ |
| *CCDC80* | X | X |  |
| *CXCL9* |  | X |  |
| *EFEMP1* | X | X |  |
| *FLRT2* |  | X |  |
| *GDF15* | X | X | X |
| *IGFBP2* |  | X | X |
| *IL15RA* |  | X |  |
| *INHBA* |  | X |  |
| *MMP12* |  | X | X |
| *NBL1* |  | X |  |
| *PRSS22* |  | X |  |
| *SMOC1* | X | X |  |
| *TFF3* |  | X |  |
| *THBS2* |  | X | X |
| *TNFRSF11B* | | X |  |
| *TNFRSF1B* |  | X |  |
| *VEGFA* |  | X |  |
| *VWC2* |  |  | X |
| *WFDC2* | X |  | X |

________________________________________________

“X” shows similar finding in one or more of the 3 studies depicted as 1, 2 and 3.

Study 1: Sathyan et al. 2020 [26]

Study 2: Tanaka et al. 2020 [27]

Study 3: Eiriksdottir et al. 2021 [28]

**References**

1. Hedrick PW Gametic disequilibrium measures: proceed with caution. Genetics. 1987;117(2):331-41.

2. Chen R, Morris BJ, Donlon TA, et al. Foxo3 longevity genotype mitigates the increased mortality risk in men with a cardiometabolic disease. Aging. 2020;12(23):23509-24.

3. Dong M, Bridges JP, Apsley K, Xu Y, Weaver TE ERdj4 and ERdj5 are required for endoplasmic reticulum-associated protein degradation of misfolded surfactant protein C. Mol Biol Cell. 2008;19(6):2620-30.

4. Bouchon A, Dietrich J, Colonna M Cutting edge: inflammatory responses can be triggered by TREM-1, a novel receptor expressed on neutrophils and monocytes. J Immunol (Baltimore). 2000;164(10):4991-5.

5. Yeh FL, Wang Y, Tom I, Gonzalez LC, Sheng M TREM2 binds to apolipoproteins, Including APOE and CLU/APOJ, and thereby facilitates uptake of amyloid-beta by microglia. Neuron. 2016;91(2):328-40.

6. Zhao Y, Wu X, Li X, et al. TREM2 Is a receptor for β-amyloid that mediates microglial function. Neuron. 2018;97(5):1023-31.e7.

7. Sudom A, Talreja S, Danao J, et al. Molecular basis for the loss-of-function effects of the Alzheimer's disease-associated R47H variant of the immune receptor TREM2. J Biol Chem. 2018;293(32):12634-46.

8. Kleinberger G, Yamanishi Y, Suárez-Calvet M, et al. TREM2 mutations implicated in neurodegeneration impair cell surface transport and phagocytosis. Science Transl Med. 2014;6(243):243ra86.

9. Filipello F, Morini R, Corradini I, et al. The microglial innate immune receptor TREM2 is required for synapse elimination and normal brain connectivity. Immunity. 2018;48(5):979-91.e8.

10. Bouchon A, Hernández-Munain C, Cella M, Colonna M A DAP12-mediated pathway regulates expression of CC chemokine receptor 7 and maturation of human dendritic cells. J Exp Med. 2001;194(8):1111-22.

11. Rajagopalan S, Long EO Cellular senescence induced by CD158d reprograms natural killer cells to promote vascular remodeling. Proc Natl Acad Sci USA. 2012;109(50):20596-601.

12. Fu B, Zhou Y, Ni X, et al. Natural killer cells promote fetal development through the secretion of growth-promoting factors. Immunity. 2017;47(6):1100-13.e6.

13. Li C, Houser BL, Nicotra ML, Strominger JL HLA-G homodimer-induced cytokine secretion through HLA-G receptors on human decidual macrophages and natural killer cells. Proc Natl Acad Sci USA. 2009;106(14):5767-72.

14. Lee N, Malacko AR, Ishitani A, et al. The membrane-bound and soluble forms of HLA-G bind identical sets of endogenous peptides but differ with respect to TAP association. Immunity. 1995;3(5):591-600.

15. Diehl M, Münz C, Keilholz W, et al. Nonclassical HLA-G molecules are classical peptide presenters. Curr Biol. 1996;6(3):305-14.

16. Rajagopalan S, Bryceson YT, Kuppusamy SP, et al. Activation of NK cells by an endocytosed receptor for soluble HLA-G. PLoS Biol. 2006;4(1):e9.

17. Gregori S, Tomasoni D, Pacciani V, et al. Differentiation of type 1 T regulatory cells (Tr1) by tolerogenic DC-10 requires the IL-10-dependent ILT4/HLA-G pathway. Blood. 2010;116(6):935-44.

18. Köstlin N, Ostermeir AL, Spring B, et al. HLA-G promotes myeloid-derived suppressor cell accumulation and suppressive activity during human pregnancy through engagement of the receptor ILT4. Eur J Immunol. 2017;47(2):374-84.

19. Rajagopalan S, Long EO A human histocompatibility leukocyte antigen (HLA)-G-specific receptor expressed on all natural killer cells. J Exp Med. 1999;189(7):1093-100.

20. Riteau B, Rouas-Freiss N, Menier C, Paul P, Dausset J, Carosella ED HLA-G2, -G3, and -G4 isoforms expressed as nonmature cell surface glycoproteins inhibit NK and antigen-specific CTL cytolysis. J Immunol (Baltimore). 2001;166(8):5018-26.

21. Naji A, Menier C, Morandi F, et al. Binding of HLA-G to ITIM-bearing Ig-like transcript 2 receptor suppresses B cell responses. J Immunol (Baltimore). 2014;192(4):1536-46.

22. Rajagopalan S, Moyle MW, Joosten I, Long EO DNA-PKcs controls an endosomal signaling pathway for a proinflammatory response by natural killer cells. Sci Signal. 2010;3(110):ra14.

23. Tilburgs T, Evans JH, Crespo Â C, Strominger JL The HLA-G cycle provides for both NK tolerance and immunity at the maternal-fetal interface. Proc Natl Acad Sci USA. 2015;112(43):13312-7.

24. Fons P, Chabot S, Cartwright JE, et al. Soluble HLA-G1 inhibits angiogenesis through an apoptotic pathway and by direct binding to CD160 receptor expressed by endothelial cells. Blood. 2006;108(8):2608-15.

25. GenCards: The human gene database. Version 5.10. 2022 <https://www.genecards.org>

26. Sathyan S, Ayers E, Gao T, et al. Plasma proteomic profile of age, health span, and all-cause mortality in older adults. Aging Cell. 2020;19(11):e13250.

27. Tanaka T, Basisty N, Fantoni G, et al. Plasma proteomic biomarker signature of age predicts health and life span. eLife. 2020;9.

28. Eiriksdottir T, Ardal S, Jonsson BA, et al. Predicting the probability of death using proteomics. Commun Biol. 2021;4(1):758.
